# Intracellular calcium dynamics permit a Purkinje neuron model to perform toggle and gain computations upon its inputs

**DOI:** 10.3389/fncom.2014.00086

**Published:** 2014-08-20

**Authors:** Michael D. Forrest

**Affiliations:** Department of Computer Science, University of WarwickCoventry, UK

**Keywords:** Purkinje, model, cerebellum, computation, sodium-potassium pump, trimodal, bimodal, toggle

## Abstract

Without synaptic input, Purkinje neurons can spontaneously fire in a repeating trimodal pattern that consists of tonic spiking, bursting and quiescence. Climbing fiber input (CF) switches Purkinje neurons out of the trimodal firing pattern and *toggles* them between a tonic firing and a quiescent state, while setting the *gain* of their response to Parallel Fiber (PF) input. The basis to this transition is unclear. We investigate it using a biophysical Purkinje cell model under conditions of CF and PF input. The model can replicate these *toggle* and *gain* functions, dependent upon a novel account of intracellular calcium dynamics that we hypothesize to be applicable in real Purkinje cells.

## Introduction

In contrast to the complexity of other brain regions, the cerebellar cortex has a single repeating connectivity motif (Ito, 1984). This motif consists of parallel, climbing, basket and stellate inputs feeding into a central Purkinje cell, which transforms them into an output. The relative simplicity of the motif makes it a good focal point for trying to understand how a brain circuit actually computes. Ultimately its computation collapses to one question, what does the Purkinje cell compute? In particular, how does its morphology and conductances transform/encode its inputs into an output? This paper addresses the issue. Forrest et al. (2012) researched the intrinsic activity of a Purkinje cell model, and how it is modulated by stellate cell input. In this study we modify this model and investigate how its intrinsic activity is modulated by climbing and parallel fiber inputs. We show that our Purkinje cell model can perform computations with these inputs, to generate an output. This is timely because there is an intense interest in the computational repertoire and power available to individual neurons (Sejnowski et al., 1988; Koch, 1999; Zador, 2000; London and Häusser, 2005; Herz et al., 2006; Mel, 2006).

*In vitro*, within cerebellar slices, Purkinje neurons can spontaneously fire action potentials in a repeating trimodal pattern that consists of tonic spiking, bursting and quiescence (Womack and Khodakhah, 2002, 2003, 2004; Womack et al., 2004; McKay and Turner, 2005; McKay et al., 2007; Forrest et al., 2012). This is upon the condition that synaptic inputs to the Purkinje cell are compromised, either by pharmacological block or the cut of the slicing plane. The repeat length of the trimodal pattern is reportedly fixed for a single Purkinje cell but has been observed to vary among different Purkinje cells, in a range from 20 s to 20 min (Womack and Khodakhah, 2002). *In vitro*, Climbing Fiber (CF) input (1 Hz) can switch a Purkinje cell out of the trimodal firing pattern and into a tonic firing pattern interrupted, at the frequency of CF input, by a complex spike and its short evoked after-pause (~20 ms long) (McKay et al., 2007). Alternatively, CF input (1 Hz) can *toggle* the Purkinje cell between a tonic firing and a quiescent state, referred to as the *up* and *down* states respectively. This toggling behavior has also been observed *in vivo* (Loewenstein et al., 2005). When the Purkinje cell is in the *up* state, CF input toggles it to the *down* state. When the Purkinje cell is in the *down* state, CF input toggles it to the *up* state. So, state transitions occur at the frequency of CF input (~1 Hz) and tonic firing periods of ~1 s alternate with quiescent periods of ~1 s [*in vitro* (McKay et al., 2007); *in vivo* (Loewenstein et al., 2005)]. How does an identical, stereotypical CF input produce opposite transitions, toggling the Purkinje cell from [*down* → *up*] and [*up* → *down*]? This is an unresolved question.

*In vitro*, Parallel Fiber (PF) input increases tonic firing frequency; CF input decreases it. In fact, CF input decreases the mean frequency of firing to a range where PF input can greatly increase it, setting the *gain* of the PF response (McKay et al., 2007).

Our Purkinje cell model intrinsically fires in the trimodal pattern (Forrest et al., 2012) and can replicate all the aforementioned responses to CF and PF input. It captures the *toggle* and *gain* computations with a novel account of intracellular Ca^2+^ dynamics, which we hypothesize to be applicable in real Purkinje cells. The model suggests how an identical CF input can produce opposite transitions, toggling the Purkinje cell from [*down* → *up*] and [*up* → *down*]. The model's *up* state responds differently to CF input than its *down* state, because it has a higher intracellular Ca^2+^ concentration in its dendrites. During tonic firing, intracellular Ca^2+^ accumulates as a function of voltage-gated Ca^2+^ entry. During quiescence it recedes as Ca^2+^ extrusion exceeds any remaining Ca^2+^ entry. A CF input event opens voltage-gated Ca^2+^ channels that pass a depolarising Ca^2+^ influx into the dendrites, which activates hyperpolarising Ca^2+^-gated SK K^+^ channels in the dendrites. So, CF input is both depolarising and hyperpolarising in parallel and which process is dominant dictates as to whether the CF input is *net* depolarising or hyperpolarising.

During the firing state, the CF conferred rise in the intracellular Ca^2+^ concentration,[Ca^2+^]_i_—added to the firing state's higher basal [Ca^2+^]_i_ value—stimulates sufficient hyperpolarising current flow (through Ca^2+^-gated SK K^+^ channels) to produce a *net* hyperpolarisation and the cell is toggled to quiescence. During the quiescent state, the CF conferred rise in [Ca^2+^]_i_—added to the quiescent state's lower basal [Ca^2+^]_i_ level—cannot confer sufficient hyperpolarising current flow (through Ca^2+^-gated SK K^+^ channels) to outweigh the depolarising effect of the Ca^2+^ entry; there is a *net* depolarisation and the cell is toggled to firing.

These model mechanisms are founded upon experimental findings: [A] In Purkinje cells, a CF input results in a marked rise in intracellular Ca^2+^ levels, which remain elevated between 1 Hz CF stimuli (Miyakawa et al., 1992; Maeda et al., 1999); [B] In Purkinje cells with high concentrations of EGTA (Ca^2+^ buffer), or with SK blocked by apamin, CF discharges are unable to block trimodal output (McKay et al., 2007).

*In vitro*, as aforementioned, repetitive CF input (1 Hz) switches a Purkinje cell out of the trimodal firing pattern and into a tonic firing pattern interrupted by either quiescent periods (~1 s long) or short pauses (~20 ms) (McKay et al., 2007). No bursting mode is observed. In both these patterns, there are very long quiescent periods (>>1 s) that seem distinct from the quiescent periods toggled by CF input (Figure 1D of McKay et al., 2007). They are punctuated by CF driven spike events at the frequency of CF input, which fail to evoke a state transition into the firing state. The cause of these long quiescent periods is not known. However, interestingly, our model can replicate them by a mechanism that we shall now explain.

The Na^+^/K^+^ pump uses the energy of one ATP molecule to exchange three intracellular Na^+^ ions for two extracellular K^+^ ions (Glitsch, 2001). Thus the pump is electrogenic, extruding one net charge per cycle to hyperpolarize the membrane potential. In our model, the quiescent mode of the trimodal pattern is produced by the electrogenic action of Na^+^/K^+^ pumping (described in detail in Forrest et al., 2012). We hypothesize that this is how real Purkinje cells produce the quiescent period of their trimodal firing pattern. Relevantly, in rat cerebellar slices, an ouabain block of Na^+^/K^+^ pumps eradicates the quiescent mode in the trimodal pattern of Purkinje cell activity, which might suggest that Na^+^/K^+^ pumping is its generative mechanism (Forrest et al., 2012).

In the model, repetitive CF input (1 Hz) blocks the bursting mode of the model's trimodal firing pattern but not its quiescent mode, generated by electrogenic Na^+^/K^+^ pumping. This enduring quiescent mode produces long quiescent periods (>>1 s) that have the same characteristics as the long quiescent periods (>>1 s) in the experimental data. Principally, they are punctuated by CF driven spike events that fail to evoke a state transition into the firing state. Extrapolating from this similarity, we suggest that the long quiescent periods in the experimental data are, as in the model, the enduring quiescent mode of the trimodal firing pattern. Furthermore, we propose that they too are generated by the electrogenic action of Na^+^/K^+^ pumping.

So, although CF input blocks the burst mode of the trimodal pattern, we suggest that Na^+^/K^+^ pump generated silences can still occur under conditions of synaptic input and we tentatively suggest that Na^+^/K^+^ pump generated silences occur physiologically. Indeed, a sizable fraction of the quiescent periods observed in Purkinje cell firing, *in vivo*, have an onset and termination that is not co-incidental or driven by CF input events (Loewenstein et al., 2005). We propose that at least some of these are Na^+^/K^+^ pump generated silences. We speculate that they have a coding function. Hence, we suggest that the Na^+^/K^+^ pump is directly involved in information processing.

## Materials and methods

Numerical simulations were performed with the NEURON 5.7 simulator (Hines and Carnevale, 1997), using its backward Euler integration method and 25 μs time steps.

Our starting point was the Purkinje cell model of Forrest et al. (2012). We took their reduced model version, a derivative of their morphologically realistic model. They produced this reduced model with a reduction algorithm (Bush and Sejnowski, 1993; Destexhe et al., 1998) that collapsed the dendritic arbor of the full model into fewer compartments, while conserving axial resistance (R_a_). This reduced model has 41 compartments as compared to 1089 in the full model. It runs significantly faster than the full model and yet faithfully reproduces its intrinsic electrical behavior (Figures 11, 12 in Forrest et al., 2012). The reduced model is made up of one soma compartment and 40 dendrite compartments: 20 of these “smooth” and 20 “spiny” (Forrest et al., 2012). The reduction algorithm used does not conserve membrane surface area. To correct for this the dendrite's membrane capacitance (*C*_*m*_) and maximal conductance values are multiplied by a correction factor, *C*_d_ = 3.80 (Forrest et al., 2012). For smooth compartments, *C*_*m*_ = 0.8 * *C*_d_ μF/cm^2^. For spiny compartments, *C*_*m*_ = 1.5 * *C*_d_ μF/cm^2^. This higher *C*_*m*_ in the spiny compartments is to represent the presence of dendritic spines.

A single EPSP/IPSP on an equivalent dendrite of the reduced model is equivalent to dividing this EPSP/IPSP and applying one fraction of it to each of the real dendrites represented by the equivalent dendrite. So, the reduced model is not appropriate for studying the effect of *single* synaptic inputs on *single* dendritic branches of Purkinje cells (e.g., studies of local dendritic processing). However, it is useful for studying the effect of *multiple* synaptic inputs that are diffuse over the dendritic tree. Indeed, the reduction algorithm employed (R_a_ conservation) has been shown to produce reduced, surrogate models that capture the synaptic integration properties of their full, parent models i.e., reduced models produced by this algorithm have been shown to perform the same non-linear integration of dendritic EPSPs and IPSPs (to have the same input-output function) as their parent models (Bush and Sejnowski, 1993).

The equations detailing our Purkinje cell model are shown later in this section. Equation 11 shows the conductances incorporated at the soma. Equation 55 shows those incorporated for the Smooth dendrite compartments. The membrane equation for a Spiny dendrite compartment (not shown) is equivalent to Equation 55, except that it is without I_SK_.

The Purkinje cell model used is nearly identical to that of Forrest et al. (2012) and like that model it intrinsically fires in the trimodal pattern of tonic spiking, bursting and quiescence. However, there are some differences. The soma compartment in the model of Forrest et al. (2012) has two, different Na^+^/K^+^ pump descriptions—one of which is highly simplified and that electrically counterbalances a highly simplified Na^+^/Ca^2+^ exchanger mechanism. Our model here only has one Na^+^/K^+^ pump description at the soma as it dispenses with the simplified Na^+^/K^+^ and Na^+^/Ca^2+^ exchanger mechanisms. It retains a realistic Na^+^/K^+^ pump description at the soma. The second disparity is that the model here, unlike that of Forrest et al. (2012), does not include an SK channel at its soma. As regards the model dendrites, here—unlike in Forrest et al. (2012)—they do not have the Kv1.2 K^+^ current, Na^+^/Ca^2+^ exchanger, Na^+^/K^+^ pump and extracellular K^+^ accumulation descriptions. Extracellular [K^+^] is fixed at 2.5 mM. The D-type K^+^ current (I_D_) description in the dendrites is modified to slow its inactivation, and the smooth dendrites' description of intracellular Ca^2+^ dynamics is reworked. A SK type Ca^2+^-activated K^+^ conductance is added to the smooth dendrites, sourced from Moczydlowski and Latorre (1983). These modifications will be explained.

In the model of Forrest et al. (2012), the tonic mode is produced by Na^+^ spiking at the soma. The burst mode is produced by the generation of Ca^2+^ spikes in the dendrites, which travel to the soma and sculpt its firing to the burst waveform. The tonic to burst transition is controlled by the Kv1.2 K^+^ current in the dendrites. This current is hyperpolarizing. It clamps dendritic excitability and prevents Ca^2+^ spike generation, which permits the tonic mode of firing. However, the power of this excitability clamp diminishes with time because K^+^ accumulates outside the dendrites and this reduces the electrochemical driving force for further K^+^ flow. Eventually the hyperpolarizing current produced by Kv1.2 channel activity is insufficient to prevent dendritic spiking and the model is switched from the tonic to the burst mode.

Kv1.2 is low-voltage gated. In addition to Kv1.2, the model has other K^+^ currents in its dendrites. However, these are largely uninvolved in the clamping of dendritic excitation as, unlike Kv1.2, they are high-voltage gated and not open at the relevant potentials. The D-type and A-type K^+^ currents are low-voltage gated but their involvement is limited because they inactivate quickly. By contrast, the Kv1.2 current is non-inactivating, which is why its current persists long enough to be tempered by slow ion relaxation processes. Indeed, experiment has shown the Kv1.2 current to be non-inactivating in the Purkinje cell (McKay et al., 2005).

During the quiescent mode of the trimodal pattern, Na^+^/K^+^ pump activity in the dendrites pumps K^+^ into the cell, reducing the extracellular K^+^ accumulation, and resets [K^+^]_o_. So, the Na^+^/K^+^ pump resets the Kv1.2 clamp to dendritic excitability and when firing resumes it is in the tonic form, not bursting.

With the stoichiometry of the Na^+^/K^+^ ATPase, for it to pump K^+^ into the dendrites it needs to simultaneously pump Na^+^ out of the dendrites (in a 2:3 ratio). The dendrites do not have voltage-dependent Na^+^ conductances (in concordance with experimental findings; Llinas and Sugimori, 1980). To permit the stereotypical operation of its Na^+^/K^+^ pumps, Na^+^ enters the dendrites through the Na^+^/Ca^2+^ exchanger.

So, in the model of Forrest et al. (2012), the tonic to burst transition is controlled by a {Kv1.2, [K^+^]_o_ accumulation, Na^+^/K^+^ pump, Na^+^/Ca^2+^ exchanger} system in the dendrites. In the model of this study, this system is absent. Its tonic to burst transition is instead controlled by the D-type K^+^ current (I_D_), which has been modified to inactivate very slowly (over seconds rather than milliseconds). This current is hyperpolarizing. It clamps dendritic excitability and prevents Ca^2+^ spike generation, which permits the tonic mode of firing. However, when this current inactivates, this excitability clamp is lost, it no longer prevents dendritic spiking and the model is switched from the tonic to the burst mode.

In this model, I_D_ is a simulacrum of the absent {Kv1.2, [K^+^]_o_ accumulation, Na^+^/K^+^ pump, Na^+^/Ca^2+^ exchanger} system in the dendrites. There is some evidence that Kv1.2 is actually the channel correlate to the I_D_ current (Shen et al., 2004). We postulate that I_D_, like Kv1.2, is non-inactivating in Purkinje cells. We confer it with slow inactivation; over the course of seconds rather than milliseconds. We do this, not to represent an inactivation *per se*, but as an abstract capture of ion relaxation. So, the same concept drives the tonic to burst transition in both models. They simply differ in their degree of abstraction of it. The additional abstraction of this model is a limitation of the study. It was introduced to make the model simpler, run faster and easier to tune. In particular, the omission of the Na^+^/Ca^2+^ exchanger from the dendrites, with its bearing on intracellular Ca^2+^ dynamics, made the manual tuning of the model's free parameters, which are principally related to intracellular Ca^2+^ dynamics, simpler.

So, the model's I_D_ current is modified from its form in Forrest et al. (2012), with an added parameter (*k* = 0.1) that slows its inactivation (refer to Equation 96 and 97). The rate of I_D_ inactivation, modulated by this *k* parameter, sets the duration of the model's tonic mode in the trimodal pattern of firing.

As aforementioned, the Purkinje cell model of this paper differs from the original model of Forrest et al. (2012) in its implementation of the [tonic → burst] transition in the trimodal pattern. However, these models do not differ in their implementation of the [burst → quiescent] and [quiescent → tonic] transitions in the trimodal pattern. In both models, the quiescent periods are generated by the electrogenic action of the Na^+^/K^+^ pump at the soma (Forrest et al., 2012). The Na^+^/K^+^ pump hyperpolarizes the membrane potential with a stoichiometry of three internal Na^+^ ions exchanged for every two external K^+^ ions. The Na^+^ concentration in a “fuzzy space” underneath the pump ([Na^+^]_i_) rises during the tonic and burst firing modes, as a function of voltage-gated Na^+^ entry. This increased intracellular Na^+^ enzymatically increases pump activity. Eventually, during the bursting mode, the pump generates such a hyperpolarizing current that firing stops and the model cell is driven to quiescence. In this case, without any spike associated Na^+^ entry, [Na^+^]_i_ would be expected to stop rising. However, [Na^+^]_i_ continues to increase as the Na^+^ influx is lagged by a parameter τ, which phenomenologically encodes the long duration of Na^+^ diffusion from the Na^+^ channel to Na^+^/K^+^ pump (Forrest et al., 2012). When τ expires, Na^+^ influx stops whilst the pump maintains Na^+^ efflux. This decreases [Na^+^]_i_, which reduces hyperpolarizing pump activity and eventually permits the model's spontaneous firing to resume. Firing resumes in the tonic spiking mode, rather than in the bursting mode which preceded the quiescence. Thus, the trimodal pattern is reset for another cycle.

In some model simulations, climbing fiber (CF) inputs were introduced. CF inputs make 17 excitatory synaptic contacts upon 17 of the model's proximal smooth dendrites (De Schutter and Bower, 1994). They fire synchronously at a frequency of 1 Hz. The CF to Purkinje synapses have a reversal potential of 0 mV, a conductance of 1 μS and their amplitude upon activation follows a dual exponential alpha function (τ_1_ = 0.5 ms; τ_2_ = 1.2 ms) (De Schutter and Bower, 1994).

In other model simulations, parallel fiber (PF) inputs were introduced. PF inputs make one excitatory synaptic contact upon each of the model's spiny dendrites (De Schutter and Bower, 1994). They fire asynchronously around a mean frequency of input (100 Hz). The PF to Purkinje synapses have a reversal potential of 0 mV, a conductance of 0.0005 μS and their amplitude upon activation follows a dual exponential alpha function (τ_1_ = 0.5 ms; τ_2_ = 1.2 ms). The Purkinje cell is known to receive ~200,000 parallel fiber synaptic contacts (Rapp et al., 1992) but our model has just 20 parallel fiber synaptic contacts (one on each spiny dendrite compartment)—0.01% of the real value. Under the conditions of random, asynchronous inputs simulated here, this missing input is compensated for by an increased firing rate of each parallel fiber synapse. A similar approach has been taken by other Purkinje cell modelers (Rapp et al., 1992; De Schutter and Bower, 1994). Assuming a linear scaling, our simulation of 0.01% of the inputs, with an asynchronous firing rate of 100 Hz, corresponds to the *realistic* average parallel fiber firing rate of ~0.01 Hz.

In some model simulations, both CF and PF inputs were introduced.

The intracellular Ca^2+^ dynamics in the spiny dendrites are as in Forrest et al. (2012). They are presented in Equations 121–123. However, the system used in the smooth dendrites is modified to be:

(1) $$\frac{d{\left[Ca^{2+}\right]}_{i}}{dt}=chan+\left(\frac{-kt*{\left[Ca^{2+}\right]}_{i}}{{\left[Ca^{2+}\right]}_{i}+kd}\right)+\left(\frac{y-{\left[Ca^{2+}\right]}_{i}}{\tau_{r}}\right)$$

(2) $$chan=\left(\frac{-(10000)*I_{Ca^{2+}}}{2*F*depth}\right)$$

(3) if(chan<0){chan=0}

(4) $$I_{Ca^{2+}}=I_{CaT}+I_{CaE}+I_{CaP}$$

Here [Ca^2+^]_i_ is the intracellular Ca^2+^ concentration in a supra-membrane shell of *depth* = 0.1 μm, *F* is the Faraday constant, *kt* = 1 * 10^−4^ mM/ms, *kd* = 1 * 10^−4^ mM, τ_r_ = 2 ms and *I*_*Ca*^2+^_ is the Ca^2+^ membrane current which is the sum of the T-type (I_CaT_), E-type (I_CaE_) and P-type (I_CaP_) voltage-gated Ca^2+^ currents. Parameter *y* is the set point Ca^2+^ concentration that the system strives to return the Ca^2+^ concentration to after a perturbation. The equation form and all of the aforementioned parameters are as in Forrest et al. (2012). However, whereas Forrest et al. (2012) has *y* specified as a constant (2.4 * 10^−4^ mM), in this model *y* is dictated by the following relationship:

(5) $$\frac{dy}{dt}=\left(\frac{I_{Ca^{2+}}/\text{​​}[d\cdot F]/4}{g}\right)+\left(\frac{z-y}{\tau_{m}}\right)$$

Here *I*_*Ca*^2+^_ is the Ca^2+^ membrane current, *F* is the Faraday constant, *d* is the compartment diameter, *z* = 2.4 * 10^−4^ mM, *g* = 1.10^5^ and τ_*m*_ = 100 ms. Constant *z* is the set point value for variable *y*. Its value is equal to that of constant *y* in Forrest et al. (2012). On the right hand side (RHS) of the equation, the first block is an intracellular Ca^2+^ accumulation term that is a modification of the intracellular Na^+^ accumulation equation in Forrest et al. (2012) (originally sourced from Canavier, 1999), with the ion changed to be Ca^2+^ and with an added parameter *g*, whose role is described later. The second block on the RHS has the same first order decay form as the third block on the RHS of Equation 1.

Equation 5 dictates that *y* increases with Ca^2+^ influx into the cell. Through Equation 1, this increase in *y* then drives increased [Ca^2+^]_i_. Studying Equations 1 and 5, it should be clear that *y* is in essence a floating equilibrium point for [Ca^2+^]_i_ that adheres to a fixed set point: *z* = 2.4 * 10^−4^ mM.

In these equations dictating [Ca^2+^]_i_: accumulation is applied to [Ca^2+^]_i_ both directly in the equation for [Ca^2+^]_i_ (first block on the RHS of Equation 1, called “chan” and given in Equation 2) and indirectly in the equation for *y* (first block on the RHS of Equation 5). The former captures [Ca^2+^]_i_ accumulation on the scale of milliseconds, during Ca^2+^/[Ca^2+^]_i_ spiking for instance, and the latter captures more gradual accumulation over seconds and minutes. So, with this layered system that uses an intermediary parameter *y*, it is possible to have [Ca^2+^]_i_ accumulation over longer time scales without disrupting the fast spiking in [Ca^2+^]_i_, This fast dendritic spiking is crucial to the Purkinje cell model because it drives somatic bursting. Anyhow, we believe that a “creeping” Ca^2+^ set point is a realistic proposition, as Ca^2+^ efflux and buffering systems are sub-linearly dependent on [Ca^2+^]_i_ because they rely on enzymes that have saturation kinetics (Stryer et al., 2002).

The *g* and τ_m_ parameters control the rate of increase (and decrease) in the Ca^2+^ set point concentration (*y*). Their value is controlled by the value of a parameter *w*:

(6) $$\begin{array}{l} \text{Initial condition},\text{ w}=0\\ \text{if }\left(I_{Ca^{2+}}>0.06{\text{mA/cm}}^{2}\right)\{w=1\}\\ \frac{dw}{dt}=\left(\frac{-w}{f}\right)f=100\text{ms}\\ \text{if }(w>0.1)\left\{g=1*10^{4},\tau_{m}=1000\right\}\\ \text{if }(w<0.1)\left\{g=1*10^{5},\tau_{m}=100\right\} \end{array}$$

The default value of *w* is 0 and so the default values of *g* and τ_*m*_ are 1 * 10^5^ and 100 respectively. However, if *I*_*Ca*^2+^_ surpasses 0.06 mA/cm^2^, as it does when there is a climbing fiber (CF) input, *w* is set to 1 and the *g* and τ_*m*_ parameters are updated to 1 * 10^4^ and 1000 respectively. The rational for these parameter changes are that the dramatic CF associated Ca^2+^ influx floods the nonlinear Ca^2+^ regulatory systems. They cannot increase their activity fast enough to match the increasing [Ca^2+^]_i_, leading to a rise in the rate of increase of [Ca^2+^]_i_. The parameter *f* sets the lifespan of the changed *g* and τ_*m*_ values by controlling the rate at which *w* attenuates. When *w* declines below 0.1, *g* and τ_*m*_ revert to their default small values. However, if there is another CF input event before *w* falls to below 0.1, *w* is reset to 1, which prolongs the lifespan of *g* and τ_*m*_ at their larger values for another cycle of *w* attenuation.

A CF input produces a Ca^2+^ spike event in the dendrites, which produces a burst event at the soma (called a *complex spike* in the literature; Kandel et al., 2000). Comparing a CF induced complex spike to a single burst from the trimodal firing pattern reveals much similarity; in experimental and model data. Why then can a complex spike toggle the Purkinje cell to quiescence and a burst of the trimodal pattern cannot? Selective coupling of calcium-activated potassium channels to specific classes of calcium channels has been experimentally observed in a number of cell types (Davies et al., 1996; Marrion and Tavalin, 1998; Smith et al., 2002; Wolfart and Roeper, 2002), including the cerebellar Purkinje cell—in two different contexts (Womack et al., 2004; Engber et al., 2012). The mechanism to this coupling is largely unknown. We extrapolate from this phenomenon to hypothesize that the dendrites have an SK type Ca^2+^-activated K^+^ conductance which is activated *only* by CF generated Ca^2+^ influx. The SK channel reads the Ca^2+^ concentration in a micro or nano domain that CF input feeds Ca^2+^ ions into. Ca^2+^ ions leave this domain, to the bulk intracellular Ca^2+^ pool, at a rate dependent on the bulk Ca^2+^ concentration. So, the CF driven accumulation of Ca^2+^ within this domain is dependent on the bulk Ca^2+^ concentration. This SK channel is not activated by the large Ca^2+^ entries that occur during the trimodal bursting mode, as this entry is not directly into its domain. It is selectively coupled to the CF input. In our model's smooth dendrites this system is realized, in an abstract manner, by the inclusion of an SK type conductance (sourced from Moczydlowski and Latorre, 1983) with a maximal conductance of g_sk_. g_sk_ is close to zero (1 * 10^−7^) at default but is assigned a higher value upon CF input. The value of g_sk_ is controlled by the value of parameter *r*:

(7) $$\begin{array}{l} \text{Initial condition}:\;r=0\\ \text{if }\left(I_{syn}>3\text{nA}\right)\{r=1\}\\ \frac{dr}{dt}=\left(\frac{-r}{s}\right)s=1000\text{ms}\\ \text{if }(r>0.1)\left\{{\text{g}}_{\text{sk}}=0.72{\text{S/cm}}^{2}\right\}\\ \text{if }(r<0.1)\left\{{\text{g}}_{\text{sk}}=1*10^{-7}{\text{S/cm}}^{2}\right\} \end{array}$$

The default value of *r* is 0 and so g_sk_ at default is 1 * 10^−7^ S/cm^2^. However, if there is CF input, the synaptic current *I*_*syn*_ across the CF synapses exceeds 3 nA and *r* is set to 1, updating g_sk_ to 0.72 S/cm^2^. The parameter *s* sets the lifespan of the changed g_sk_ value by controlling the rate at which *r* attenuates. When *r* declines below 0.1, g_sk_ reverts to its default value of 1 * 10^−7^ S/cm^2^. However, if there is another CF input event before *r* falls to below 0.1, *r* is reset to 1, which prolongs the lifespan of g_sk_ at its higher value for another cycle of *r* attenuation.

This system of intracellular Ca^2+^ dynamics permits CF input to *toggle* the Purkinje cell model between a firing and a quiescent state (*refer to Results*). It is an extremely abstract description, but it is founded upon good rationale in the absence of conclusive experimental data—our present understanding of intracellular Ca^2+^ dynamics is extremely limited and so there is not much to guide its modeling. Available data does not constrain the free parameters of the model: *g*, τ_*m*_, *f, w, z, g*_sk_, *r, s*. These were tuned manually (Prinz, 2006) by iteratively running the model with different free parameter values and observing which combination of these gave the best fit between real and model Purkinje cell output. Aside from these parameters, the Purkinje cell model's current, synapse and pump equations were predominantly parameterized to experimental data as detailed in Forrest et al. (2012). Although our model has a reduced morphology, and can run faster than the full model of Forrest et al. (2012), it is still quite computationally expensive in absolute terms. With CF input, 51 s of CPU time is required for 1 s of Purkinje cell simulation on an Intel Pentium PC (I5). A typical simulation would observe Purkinje cell behavior over 40 s and would require ~34 min of CPU time. This cost hindered model tuning as it adversely delimited the number of model runs per unit time. It was another factor that constrained the complexity of our implemented intracellular Ca^2+^ dynamics. The abstract nature of the model's intracellular Ca^2+^ system is a limitation of this study. In time, as experiments parse more detail, and desktop computers become more powerful, we hope that our model can be improved.

### The equations of the purkinje cell model

*C*_*m*_ is the membrane capacitance, *I* is current, *V* is the membrane potential in mV as a dimensionless quantity, *t* is time, *T* is temperature (36°C), *Ra* is the specific axial resistivity (35.4 Ω cm), *F* is the Faraday constant, *R* is the gas constant and g_max_ is the maximal conductance. g_max_ values, for the different currents, are shown in Table 1.

**Table 1:** **Maximal conductances (g_max_; mS/cm^2^)**.

**Current**	**Soma**	**Dendrite**
Resurgent Na^+^	156	0
P-type Ca^2+^	0.52	6.1
T-type Ca^2+^	0	2.28
E-type Ca^2+^	0	12.16
A-type K^+^	0	121.6
D-type K^+^	0	136.8
M-type K^+^	0	0.0152
Delayed rectifier K^+^	0	0.912
Bk K^+^	72.8	228
K2 K^+^	0	0.608
Highly TEA sensitive K^+^	41.6	0
Moderately TEA sensitive K^+^	20.8	0
TEA insenstitive K^+^	41.6	0
Hyperpolarization activated cation, I_*h*_	1.04	1.4
Leak	0.1	0.38

*The smooth dendrite compartments do not have a SK-type K^+^ conductance but the Spiny dendrite compartments can express such a conductance (g_max_ = 0.72 or 0.62 S/cm^2^) on the condition of CF-input, as explained in Methods*.

*m, h* and *z* are Hodgkin-Huxley “particles”/gates (Hille, 2001); for example, for the *m* Hodgkin-Huxley gate:

(8) $$\frac{dm}{dt}=\frac{m_{\infty }-m}{\tau_{m}}$$

The voltage (and/or intracellular Ca^2+^) dependence of a Hodgkin–Huxley (H–H) current (Hille, 2001) can be expressed by stating, for each H–H gate (e.g., for the *m* gate), either [*m*_∞_, τ_*m*_] OR [α_*m*_, β_*m*_]. These entities are voltage (and/or intracellular Ca^2+^) dependent. The latter set can give the former set through the relations:

(9) $$m_{\infty }=\text{​}\alpha_{m}/\left(\alpha_{m}+\beta_{m}\right)$$

(10) $$\tau_{m}=1\text{​}\text{​}/\left(\alpha_{m}+\beta_{m}\right)$$

### Soma

The soma is a cylinder (length = 22 μm, diameter = 22 μm). *C*_*m*_ = 0.8 μF/cm^2^. The soma has highly TEA sensitive (I_K_fast_), moderately TEA sensitive (I_K_mid_) and TEA insensitive (I_K_slow_) voltage-gated K^+^ currents, a BK voltage-and-Ca^2+^-gated K^+^ current (I_BK_), a resurgent Na^+^ current (I_Na−R_), a P-type Ca^2+^ current (I_CaP_), a hyperpolarization activated cation current (I_H_), a leak current (I_L_), a Na^+^/K^+^ pump (i^s^_pump_), an intracellular Ca^2+^ dynamics description and an account of intracellular Na^+^ accumulation. It receives a current from, or sends a current to, its connecting, adjacent dendrite compartment (I_transfer_DS_). The membrane equation for the soma:

(11) $$\begin{array}{l} C_{m}\cdot \frac{dV}{dt}=-(I_{K\_fast}+I_{K\_mid}+I_{K\_slow}+I_{BK}+I_{CaP}\\ \;+I_{H}+I_{L}+I_{NaR}+i_{pump}^{s}+I_{transfer\_DS}) \end{array}$$

E_K_ is the reversal potential for K^+^ (initiated at −88 mV), E_Na_ is the reversal potential for Na^+^(initiated at +70 mV), E_Ca_ is the reversal potential for Ca^2+^(initiated by the NEURON default value; +132 mV), E_L_ is the reversal potential for the Leak current (−70 mV), E_h_ is the reversal potential for the hyperpolarisation activated cation current (−30 mV), Intracellular Ca^2+^ concentration is initiated at the NEURON default of 5e^−5^ mM; Extracellular Ca^2+^ concentration is initiated at the NEURON default of 2 mM. The somatic membrane voltage (*V*) is initiated at the NEURON default of −65 mV.

### Dendrite—soma electrotonic current (Carnevale and Hines, 2006)

(12) $$I_{transfer\_DS}=\frac{\left(V_{D}-V_{S}\right)}{R_{DS}}$$

(13) $$R_{DS}=\frac{Ra\cdot (L_{S}/2)}{\pi \cdot r_{S}^{2}}+\frac{Ra\cdot (L_{D}/2)}{\pi \cdot r_{D}^{2}}$$

V_D_ is the membrane voltage at the center of the dendrite compartment, V_S_ is the membrane voltage at the center of the soma compartment and R_DS_ is the axial Resistance between the two. Ra is the specific axial Resistivity, L_S_ and r_S_ are the length and radius of the soma respectively; L_D_ and r_D_ are the length and radius of the dendrite compartment respectively.

### Highly TEA sensitive K^+^ current (Khaliq et al., 2003)

(14) $$I_{K\_fast}=g_{\max}\cdot m^{3}\cdot h\cdot (V-E_{K})$$

(15) $$m_{\infty }=\frac{1}{\exp\left(-\frac{V--24}{15.4}\right)}$$

(16) $$\tau_{m}=\{\begin{array}{l} 0.000103+0.0149*\exp(0.035*V).......\\ \;[V<-35mV]\\ 0.000129+1/\left[\exp\text{​}\left(\frac{V+100.7}{12.9}\right)+\exp\left(\frac{V-56}{-23.1}\right)\right]......\\ \;[V\geq -35mV] \end{array}$$

(17) $$h_{\infty }=0.31+\frac{1-0.31}{\exp\left(-\frac{V--5.8}{-11.2}\right)}$$

(18) $$\tau_{h}=\{\begin{array}{l} 1.22*10^{-5}+0.012*\exp\left[-{\left(\frac{V+56.3}{49.6}\right)}^{2}\right]........\\ \;[V\leq 0mV]\\ 0.0012+0.0023*\exp(-0.141*V).........\\ \;[V>0mV] \end{array}$$

### Moderately TEA sensitive K^+^ current (Khaliq et al., 2003)

(19) $$I_{K\_mid}=g_{\max}\cdot m^{4}\cdot (V-E_{K})$$

(20) $$m_{\infty }=\frac{1}{\exp\left(-\frac{V--24}{20.4}\right)}\;$$

(21) $$\tau_{m}=\{\begin{array}{l} 0.000688+1/\left[\exp\left(\frac{V+64.2}{6.5}\right)+\exp\left(\frac{V-141.5}{-34.8}\right)\right].....\\ \;[V<-20mV]\\ 0.00016+0.0008*\exp(-0.0267*V)........\\ \;[V\geq -20mV] \end{array}$$

### TEA insensitive K^+^ current (Khaliq et al., 2003)

(22) $$I_{K\_slow}=g_{\max}\cdot m^{4}\cdot (V-E_{K})$$

(23) $$m_{\infty }=\frac{1}{\exp\left(-\frac{V--16.5}{18.4}\right)}$$

(24) $$\begin{array}{l} \tau_{m}=0.000796+1/\\ \;\left[\exp\left(\frac{V+73.2}{11.7}\right)+\exp\left(\frac{V-306.7}{-74.2}\right)\right] \end{array}$$

### P-type Ca^2+^ current (Khaliq et al., 2003)

(25) $$I_{CaP}=g_{\max}*m*ghk$$

Goldman–Hodgkin–Katz (ghk) equation:

(26) $$\begin{array}{l} ghk=\left(4*P_{Ca^{2+}}\right)*\frac{V\cdot F^{2}}{R\cdot T}\\ \;*\frac{{\left[Ca^{2+}\right]}_{i}-{\left[Ca^{2+}\right]}_{o}*\exp\text{​}\left(\frac{-2\cdot F\cdot V}{R\cdot T}\right)}{1-\exp\left(\frac{-2\cdot F\cdot V}{R\cdot T}\right)} \end{array}$$

P^2+^_*Ca*_ is 5 * 10^−5^ cm/sec, [Ca^2+^]_i_ = 100 nM, [Ca^2+^]_o_ = 2 mM, T = 295 K, F is the Faraday constant and R is the gas constant. [Ca^2+^]_i_ and [Ca^2+^]_o_ are fixed constants, as seen by this equation—it does *not* access the changing value of [Ca^2+^]_i_ as set by the intracellular Ca^2+^ equations (given later).

(27) $$m_{\infty }=\frac{1}{\exp\left(-\frac{V--19}{5.5}\right)}$$

(28) $$\tau_{m}=\{\begin{array}{l} 0.000264+0.128*\exp(0.103*V)...............\\ \;[V\leq -50mV]\\ 0.000191+0.00376*\exp\left[-{\left(\frac{V+11.9}{27.8}\right)}^{2}\right].......\\ \;[V>-50mV] \end{array}$$

### Hyperpolarisation activated cation current (Khaliq et al., 2003)

(29) $$I_{H}=g_{\max}\cdot m\cdot (V-E_{h})$$

(30) $$m_{\infty }=\frac{1}{\exp\left(-\frac{V--90.1}{-9.9}\right)}$$

(31) $$\tau_{m}=0.19+0.72*\exp\left[-{\left(\frac{V+81.5}{11.9}\right)}^{2}\right]$$

### BK type K^+^ current (Khaliq et al., 2003)

(32) $$I_{BK}=g_{\max}\cdot m^{3}\cdot z^{2}\cdot h\cdot (V-E_{K})$$

(33) $$m_{\infty }=\frac{1}{\exp\left(-\frac{V--28.9}{6.2}\right)}$$

(34) $$h_{\infty }=0.085+\frac{1-0.085}{\exp\left(-\frac{V--32}{-5.8}\right)}$$

(35) $$\begin{array}{l} \tau_{m}=0.000505\\ \;+1/\left[\exp\left(\frac{V+86.4}{10.1}\right)+\exp\left(\frac{V-33.3}{-10}\right)\right] \end{array}$$

(36) $$\begin{array}{l} \tau_{h}=0.0019\\ \;+1/\left[\exp\left(\frac{V+48.5}{5.2}\right)+\exp\left(\frac{V-54.2}{-12.9}\right)\right] \end{array}$$

(37) $$z_{\infty }=\frac{1}{1+\frac{0.001}{\left[Ca^{2+}\right]}}$$

(38) $$\tau_{z}=1$$

### Leak current (Khaliq et al., 2003)

(39) $$I_{L}=g_{\max}*(V-E_{L})$$

### Intracellular Ca^2+^ concentration (Khaliq et al., 2003)

[Ca^2+^] is calculated for the intracellular space within 100 nm of the membrane. [Ca^2+^] changes as I^2+^_*Ca*_ (negative by convention; inward currents are negative) brings Ca^2+^ into this space and as Ca^2+^ leaves by diffusion to the bulk cytoplasm. The diffusion rate constant, β, is set to 1/ms.

(40) $$\frac{d\left[Ca^{2+}\right]}{dt}=\beta *[Ca^{2+}]$$

[Ca^2+^] at time step, *t*:

(41) $$\begin{array}{l} {\left[Ca^{2+}\right]}_{t}={\left[Ca^{2+}\right]}_{t-1}\\ \;+\Delta t*\left(\frac{-(100)*I_{Ca^{2+}}}{\left(2\cdot F)*(depth\cdot Area\right)}-\beta *{\left[Ca^{2+}\right]}_{t-1}\right) \end{array}$$

*F* is the Faraday constant, *depth* = 0.1 μm and membrane surface *Area* = 1521μm^2^. [Ca^2+^] was constrained to not fall below 100 nM by coding of the form:

(42) $$if\;([Ca^{2+}]<100)\left\{[Ca^{2+}]=100\right\}$$

### Resurgent Na^+^ current (Khaliq et al., 2003)

(43) $$\begin{array}{l} I_{NaR}=\underset{\max}{g}*O*(V-E_{Na})\\ O\text{ is the occupancy of the Open state}. \end{array}$$

**Figure 1:**
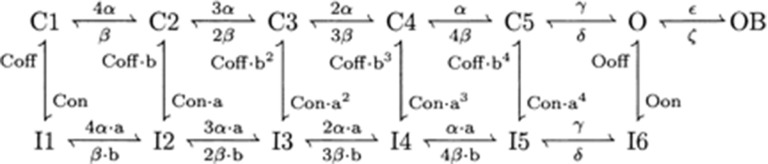
**The Resurgent Na^+^ current is described by a Markov scheme (Raman and Bean, 2001; Khaliq et al., 2003)**. (C1–C5) denote sequential Closed states; O denotes the Open state. (I1–I6) denote Inactivated states. OB denotes the state entered by a second mechanism of inactivation, which is hypothesized to be equivalent to Open Channel Block. The rate constants between states are given in Equation [44–49].

This current is described by a Markov scheme, shown in Figure 1. The rate constants, labeled in Figure 1, are (ms^−1^):

(44) $$\alpha =150*\exp\left(\frac{V}{20}\right)$$

(45) $$\beta =3*\exp\left(\frac{2\cdot V}{20}\right)$$

$$\begin{array}{l} \gamma =150;\delta =40;\;Con=0.005;\;Coff=0.5;\;Oon=0.75;\\ Ooff=0.005 \end{array}$$

(46) $$a={\left(\frac{Oon}{Con}\right)}^{1/4}$$

(47) $$b={\left(\frac{Ooff}{Coff}\right)}^{1/4}$$

(48) ε=1.75

(49) $$\zeta =0.03*\exp\left(\frac{2\cdot V}{25}\right)$$

### Na^+^/K^+^ pump (Forrest et al., 2012)

The somatic Na^+^/K^+^ pump (density = d^s^_pump_, 0.04 mA/cm^2^) transports 3 Na^+^ out (i^s^_pump_Na_) for every 2 K^+^ in (i^s^_pump_K_). It has an exponential relation to intracellular Na^+^ concentration ([Na^+^]_i_).

(50) $$\{\begin{array}{l} {\text{i}}_{\text{pump}}^{\text{s}}={\text{d}}_{\text{pump}}^{\text{s}}/\text{​​}\left(1+\text{exp}\left[\frac{40-{\left[{\text{Na}}^{+}\right]}_{\text{i}}}{1}\right]\right)\\ {\text{i}}_{\text{pump}\_\text{Na}}^{\text{s}}=3{\text{i}}_{\text{pump}}^{\text{s}}\\ {\text{i}}_{\text{pump}\_\text{K}}^{\text{s}}=-2{\text{i}}_{\text{pump}}^{\text{s}} \end{array}$$

### Intracellular Na^+^ concentration (Forrest et al., 2012)

[Na^+^]_i_ is initiated at 10 mM and then changes in time,

(51) $$\frac{\partial {\left[Na^{+}\right]}_{i}}{\partial t}=\frac{I_{Na\_net}}{[d\cdot F\cdot (10000)]/4}+\frac{D\partial^{2}{\left[Na^{+}\right]}_{i}}{{\left(\partial x\right)}^{2}}$$

(52) $${\left(I_{Na\_net}\right)}_{\left[t\right]}={\left(I_{Na\_R}-i_{pump\_Na}\right)}_{\left[t-\tau \right]},\;\tau =5s$$

*F* is the Faraday constant, *d* is the somatic diameter and *D* is the diffusivity constant (0.6 μm^2^/ms). The second term on the RHS of Equation 51 accounts for longitudinal diffusion of Na^+^ out of the soma compartment, along the longitudinal distance (*x*). The effects of this term are fairly negligible and it can be dropped to quicken simulation speeds. *I*_*Na_net*_ is the difference between Na^+^ current flowing into the soma (through *I*_*Na−R*_) and Na^+^ current pumped out of the soma by the Na^+^/K^+^ pump (*i*_pump_Na_), lagged by parameter τ = 5 s. Intracellular Na^+^ stimulates the Na^+^/K^+^ pump and this lag τ accounts for the duration of sodium's diffusion from channels to pumps. “Catch coding” is applied:

(53) $$\text{if }({\left[{\text{Na}}^{+}\right]}_{\text{i}}<10)\left[{\left[{\text{Na}}^{+}\right]}_{\text{i}}=10\right]$$

(54) $$\text{if }({\text{E}}_{\text{Na}}<70)[{\text{E}}_{\text{Na}}=70]$$

### Dendrite

The model's dendrite projection is split into 40 compartments; 20 of these “smooth” and 20 “spiny” (Forrest et al., 2012). The model's dendrite correction factor: *C*_*d*_ = 3.80 (Forrest et al., 2012). For smooth compartments, *C*_*m*_ = 0.8 * *C*_*d*_ μF/cm^2^. For spiny compartments, *C*_*m*_ = 1.5 * *C*_*d*_ μF/cm^2^.

All the mechanisms in the dendrite are distinct from those in the soma. The dendrite has hyperpolarization activated cation current (I_H_); T-type (I_CaT_), Class-E (I_CaE_) and P-type (I_CaP_) voltage-gated Ca^2+^ currents; a leak current (I_L_); A-type (I_KA_), D-type (I_KD_), M-type (I_KM_) and Delayed Rectifier (I_DR_) voltage-gated K^+^ currents; BK (I_BK_) and K2 (I_K2_) type voltage-and-Ca^2+^-gated K^+^ currents and an intracellular Ca^2+^ dynamics description.

In addition to the aforementioned, Smooth compartments have an SK-type K^+^conductance (I_SK_) and a different intracellular Ca^2+^ dynamics description than that incorporated in Spiny compartments. Aside from these disparities, and their differing *C*_*m*_ value, Smooth and Spiny compartments are intrinsically equivalent. They receive different synaptic inputs: CF inputs project to Smooth, PF inputs project to Spiny. These inputs were described earlier. The membrane equation for a Smooth compartment, with its additional I_SK_ conductance:

(55) $$C_{m}\cdot \frac{dV}{dt}=-\left(\begin{array}{l} I_{CaT}+I_{CaE}+I_{CaP}+I_{H}+I_{KA}+I_{KM}\\ +I_{KD}+I_{DR}+I_{BK}+I_{K2}+I_{L}+I_{SK}\\ +I_{trans\_one}+I_{trans\_two} \end{array}\right)$$

*I*_*trans_one*_ is the current from a neighboring compartment. *I*_*trans_two*_ is the current from the other neighboring compartment. These currents follow the same form as that described for the transfer between the soma and its adjacent 1st dendrite compartment in Equation 12.

E_K_ is the reversal potential for K^+^ (initiated by the NEURON default value; −77 mV), E_Na_ is the reversal potential for Na^+^ (initiated by the NEURON default value; +50 mV), E_Ca_ is the reversal potential for Ca^2+^ (initiated by the NEURON default value; +132 mV), E_L_ is the reversal potential for the Leak current (−80 mV), E_h_ is the reversal potential for the hyperpolarisation activated cation current (−32.9 mV). Intracellular Ca^2+^ concentration is initiated at 4e^−5^ mM; Extracellular Ca^2+^ concentration is initiated at 2.4 mM.

### T-type Ca^2+^ current (Miyasho et al., 2001)

(56) $$\begin{array}{l} I_{CaT}=g_{\max}\cdot m\cdot h\cdot (V-E_{Ca});\\ {\text{E}}_{\text{Ca}}\text{ is fixed at }+135\text{ mV for this current}. \end{array}$$

(57) $$\alpha_{m}=\frac{2.6}{1+\exp\left(\frac{V+21}{-8}\right)}$$

(58) $$\beta_{m}=\frac{0.18}{1+\exp\left(\frac{V+40}{4}\right)}$$

(59) $$\alpha_{h}=\frac{0.0025}{1+\exp\left(\frac{V+40}{8}\right)}$$

(60) $$\beta_{h}=\frac{0.19}{1+\exp\left(\frac{V+50}{-10}\right)}$$

$mt=3^{\frac{T-37}{10}}$; *T* is temperature in degrees centigrade (36).

(61) $$\tau_{m}=\frac{1}{\left(\alpha_{m}+\beta_{m}\right)\cdot mt}$$

(62) $$\tau_{h}=\frac{1}{\left(\alpha_{h}+\beta_{h}\right)\cdot mt}$$

### E-type Ca^2+^ current (Miyasho et al., 2001)

(63) $$\begin{array}{l} I_{CaE}=g_{\max}\cdot m\cdot h\cdot (V-E_{Ca});\\ {\text{E}}_{\text{Ca}}\text{ is fixed at }+135\text{mV for this current}. \end{array}$$

(64) $$\alpha_{m}=\frac{2.6}{1+\exp\left[\left(V+7\right)/-8\right]}$$

(65) $$\beta_{m}=\frac{0.18}{1+\exp\left[\left(V+26\right)/4\right]}$$

(66) $$\alpha_{h}=\frac{0.0025}{1+\exp\left[\left(V+32\right)/8\right]}$$

(67) $$\beta_{h}=\frac{0.19}{1+\exp\left[\left(V+42\right)/-10\right]}$$

$mt=3^{\frac{T-37}{10}}$; *T* is temperature in degrees centigrade (36).

(68) $$m_{\exp}=1-\exp\left(\frac{\left[-dt*mt]\cdot [\alpha_{m}+\beta_{m}\right]}{4}\right)$$

(69) $$h_{\exp}=1-\exp\left(\frac{\left[-dt*mt]\cdot [\alpha_{h}+\beta_{h}\right]}{10}\right)$$

### P-type Ca^2+^ current (Miyasho et al., 2001)

(70) $$\begin{array}{l} I_{CaP}=g_{\max}\cdot m\cdot (V-E_{Ca});\\ {\text{E}}_{\text{Ca}}\text{ is fixed at }+135\text{mV for this current}. \end{array}$$

(71) $$\alpha_{m}=\frac{8.5}{1+\exp\left(\left[V+-8\right]/-12.5\right)}$$

(72) $$\beta_{m}=\frac{35}{1+\exp\left(\left[V+74\right]/14.5\right)}$$

$mt=3^{\frac{T-37}{10}}$; *T* is temperature in degrees centigrade (36).

(73) $$\tau_{m}=\frac{1}{\left(\alpha_{m}+\beta_{m}\right)\cdot mt}$$

### Hyperpolarisation activated cation current (Saraga et al., 2003)

(74) $$I_{h}=g_{\max}\cdot m\cdot (V-E_{h});{\text{E}}_{h}=-32.9\text{mV}$$

(75) $$\tau_{m}=\frac{1}{\begin{array}{l} \exp(-17.9-0.116\cdot V)\\ \;+\exp(-1.84+0.09\cdot V) \end{array}}+100$$

(76) $$m_{\infty }=\frac{1}{1+\exp[(V+84.1)/10.2]}$$

(77) $$m_{\exp}=1-\exp\left(\frac{-dt}{\tau_{m}}\right)$$

### A-type K^+^ current (Miyasho et al., 2001)

(78) $$I_{KA}=g_{\max}\cdot m^{4}\cdot h\cdot (V-E_{K})$$

(79) $$\alpha_{m}=\frac{1.4}{1+\exp([V+27]/-12)}$$

(80) $$\beta_{m}=\frac{0.49}{1+\exp([V+30/4)}$$

(81) $$\alpha_{h}=\frac{0.00175}{1+\exp([V+50/8)}$$

(82) $$\beta_{h}=\frac{0.49}{1+\exp([V+13/-10)}$$

$mt=3^{\frac{T-37}{10}}$; *T* is temperature in degrees centigrade (36).

(83) $$\tau_{m}=\frac{1}{\left(\alpha_{m}+\beta_{m}\right)\cdot mt}$$

(84) $$\tau_{h}=\frac{1}{\left(\alpha_{h}+\beta_{h}\right)\cdot mt}$$

### M-type K^+^ current (Miyasho et al., 2001)

(85) $$I_{KM}=g_{\max}\cdot m\cdot (V-E_{K})$$

$ft=2.3^{\frac{T-36}{10}}$; *T* is temperature in degrees centigrade (36).

(86) $$\tau_{m}=\frac{1000/ft}{3.3\cdot \left(e^{+(V+35)/40}+e^{-(V+35)/20}\right)}$$

(87) $$m_{\infty }=\frac{1}{1+e^{-(V+35)/10}}$$

### D-type K^+^ current (Miyasho et al., 2001)

(88) $$I_{KD}=g_{\max}\cdot m\cdot h\cdot (V-E_{K})$$

(89) $$\alpha_{m}=\frac{8.5}{1+\exp([V+17]/-12.5)}$$

(90) $$\beta_{m}=\frac{35}{1+\exp([V+99]/14.5)}$$

(91) $$\alpha_{h}=\frac{0.0015}{1+\exp([V+89]/8)}$$

(92) $$\beta_{h}=\frac{0.0055}{1+\exp([V+83]/-8)}$$

(93) $$m_{\infty }=\alpha_{m}/\text{​}\left(\alpha_{m}+\beta_{m}\right)$$

(94) $$h_{\infty }=\alpha_{h}/\text{​}\left(\alpha_{h}+\beta_{h}\right)$$

$mt=3^{\frac{T-37}{10}}$; *T* is temperature in degrees centigrade (36).

(95) $$m_{\exp}=1-\exp\left(\frac{\left[-dt*mt]\cdot [\alpha_{m}+\beta_{m}\right]}{10}\right)$$

(96) $$h_{\exp}=1-\exp\left([-dt*mt]\cdot [\alpha_{h}+\beta_{h}]\cdot k\right)$$

(97) k=0.1

### Delayed rectifier type K^+^ current (Miyasho et al., 2001)

(98) $$I_{DR}=g_{\max}\cdot m^{4}\cdot (V-E_{K})$$

(99) $$\alpha_{m}=0.1\cdot vtrap$$

(100) $$catch=fabs\left(\frac{-(V+55)}{10}\right)$$

Where fabs(x) returns the absolute value of a floating point number; the absolute value of its argument (|x|).

(101) $$vtrap=\{\begin{array}{l} 10\cdot \left(1-\frac{-(V+55)}{10}/2\right)....................\\ \;[catch<1e^{-6}]\\ \frac{-(V+55)}{\exp\left(\frac{-(V+55)}{10}\right)-1}.......................\\ \;[catch\geq 1e^{-6}] \end{array}\;$$

(102) $$\beta_{m}=0.125\cdot \exp\left(\frac{-(V+65)}{80}\right)$$

$mt=3^{\frac{T-37}{10}}$; *T* is temperature in degrees centigrade (36).

(103) $$m_{\exp}=1-\exp\left(-dt\cdot mt\cdot \left[\alpha_{m}+\beta_{m}\right]\right)$$

### BK-type K^+^ current (Miyasho et al., 2001)

(104) $$I_{BK}=g_{\max}\cdot m\cdot z^{2}\cdot (V-E_{K})$$

(105) $$\alpha_{m}=7.5$$

(106) $$\beta_{m}=\frac{0.11}{\exp\left(\left[V+-35\right]/14.9\right)}$$

(107) $$m_{\exp}=1-\exp\left(-dt\cdot [\alpha_{m}+\beta_{m}]\right)$$

(108) $$\alpha_{z}=1$$

(109) $$\beta_{z}=\frac{400}{[Ca^{2+}]*1000}$$

(110) $$\tau_{z}=10$$

(111) $$z_{\exp}=1-\exp\left(\frac{-dt}{\tau_{z}}\right)$$

### K2-type K^+^ current (Miyasho et al., 2001)

(112) $$I_{K2}=g_{\max}\cdot m\cdot z^{2}\cdot (V-E_{K})$$

(113) $$\alpha_{m}=25$$

(114) $$\beta_{m}=\frac{0.075}{\exp\left(\left[V+5\right]/10\right)}$$

(115) $$m_{\exp}=1-\exp\left(-dt\cdot [\alpha_{m}+\beta_{m}]\right)$$

(116) $$\alpha_{z}=1$$

(117) $$\beta_{z}=\frac{20}{[Ca^{2+}]*1000}$$

(118) $$\tau_{z}=10$$

(119) $$z_{\exp}=1-\exp\left(\frac{-dt}{\tau_{z}}\right)$$

### Leak current (Miyasho et al., 2001)

(120) $$\begin{array}{l} I_{L}=g_{\max}*(V-E_{L});\\ {\text{E}}_{\text{L}}\text{ is }-80\text{mV for this current in the dendrite}. \end{array}$$

### Intracellular Ca^2+^ dynamics in a spiny dendrite compartment (Miyasho et al., 2001)

(121) $$\begin{array}{l} \frac{d{\left[Ca^{2+}\right]}_{i}}{dt}=chan+\left(\frac{-kt*{\left[Ca^{2+}\right]}_{i}}{{\left[Ca^{2+}\right]}_{i}+kd}\right)\\ \;+\left(\frac{y-{\left[Ca^{2+}\right]}_{i}}{tau_{r}}\right) \end{array}$$

(122) $$chan=\left(\frac{-(10000)*I_{Ca^{2+}}}{2*F*depth}\right)$$

(123) if(chan<0){chan=0}

Where [Ca^2+^]_i_ is the intracellular Ca^2+^ concentration in a supra-membrane shell of *depth* = 0.1 μm, *F* is the Faraday constant, *I*_*Ca*^2+^_ is the Ca^2+^ membrane current (negative by convention; inward currents are negative), *kt* = 4e^−5^ mM/ms, *kd* = 4e^−5^ mM, tau_*r*_ = 2 ms and y = 4e^−5^ mM.

### Intracellular Ca^2+^ dynamics in a smooth dendrite compartment

This was described earlier with Equations 1–6.

### SK-type K^+^ current (incorporated in smooth and not spiny dendrite compartments) (Moczydlowski and Latorre, 1983)

(124) $$I_{SK}=g_{\max}\cdot m\cdot (V-E_{K})$$

(125) $$\alpha_{m}=\frac{0.48}{1+\left(0.18\cdot \exp\left(\frac{-2\cdot 0.84\cdot F\cdot V/R}{T+273.15}\right)\right)/[Ca^{2+}]}$$

(126) $$\beta_{m}=\frac{0.28}{1+[Ca^{2+}]/\left(0.011\cdot \exp\left[\frac{-2\cdot 1\cdot F\cdot V/R}{T+273.15}\right]\right)}$$

(127) $$\tau_{m}=\frac{1}{\alpha_{m}+\beta_{m}}$$

(128) $$m_{\infty }=\frac{\alpha_{m}}{\alpha_{m}+\beta_{m}}$$

g_max_ for this current is not a fixed constant but is varied as described with Equation 7.

## Results

### Parallel fiber (PF) input changes the mean frequency, but not the pattern, of purkinje cell firing in the model

**Figure 2:**
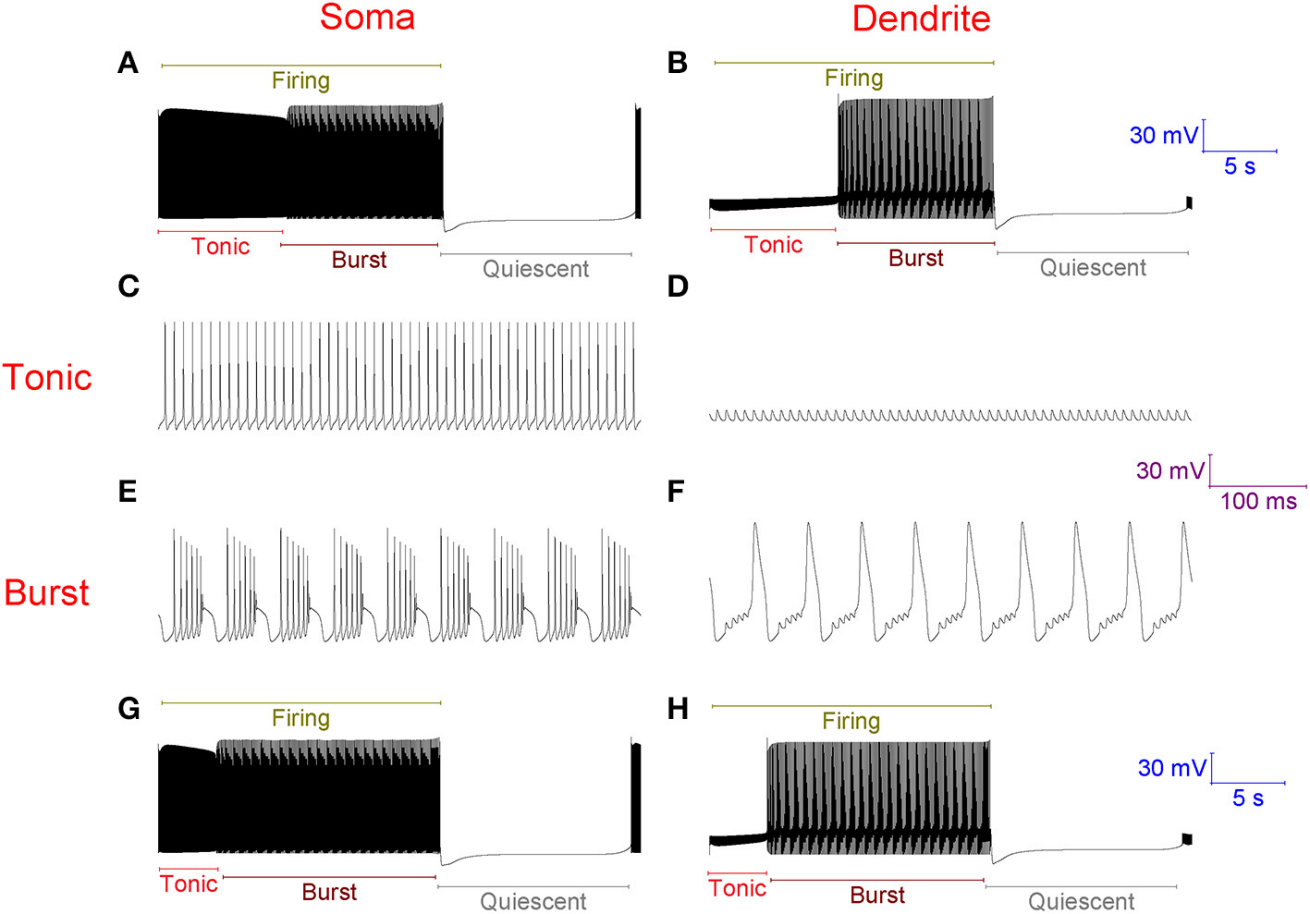
**Without synaptic innervation, the Purkinje cell model intrinsically fires in a repeating trimodal pattern of tonic spiking, bursting and quiescence**. **(A)**, Single trimodal repeat, recorded at the soma, with the constituent tonic, burst and quiescent modes labeled. The firing length (tonic+burst) is also labeled. The trimodal repeat length is ~20 s, which is within the experimentally reported range (Womack and Khodakhah, 2002). **(B)** The same trimodal repeat as **(A)** but recorded from a point within the dendritic tree. One can compare panels **(A,B)** and observe that with no high-threshold spikes in the dendrites, firing is tonic at the soma. With high-threshold dendritic spikes, bursting occurs at the soma. **(C)** Tonic firing at the soma, shown at high resolution. **(D)** The same timeframe as **(C)**, but recorded from a point within the dendritic tree. **(E)** Burst firing at the soma, shown at high resolution; it has a very stereotypical waveform. F, The same timeframe as **(E)**, but recorded from a point within the dendritic tree. **(G)** Single trimodal repeat, recorded at the soma, when the model parameter *k* is 0.2, instead of the model's default value of 0.1. *k* is a parameter controlling the rate of inactivation to the I_D_ K^+^ current, and this rate controls the timing of the tonic to burst transition (*refer to Methods*). With this modified *k* value, the tonic length is shorter than in **(A)**; a greater proportion of the firing length is occupied by bursting. **(H)** The same trimodal repeat as panel **(G)** but recorded from a point within the dendritic tree. **(A,B)** Are scaled by the first scale bar (30 mV, 5 s); **(C–F)** are scaled by the second scale bar (30 mV, 100 ms); **(G,H)** are scaled by the third scale bar (30 mV, 5 s).

With no synaptic innervation, the Purkinje cell model intrinsically fires in a repeating trimodal pattern of tonic spiking, bursting and quiescence (Figure 2). Without high-threshold Ca^2+^ spikes in the dendrites (Figure 2D), firing is tonic at the soma (Figure 2C). With high-threshold dendritic Ca^2+^ spikes (Figure 2F), bursting occurs at the soma (Figure 2E). The tonic to burst transition is controlled by the hyperpolarizing D-type K^+^ current (I_D_) in the dendrites. This current is hyperpolarizing. It clamps dendritic excitability and prevents Ca^2+^ spike generation, which permits the tonic mode of firing. However, when this current inactivates, this excitability clamp is lost, it no longer prevents dendritic spiking and the model is switched from the tonic to the burst mode. The *k* model parameter controls the rate of I_D_ inactivation (*refer to Methods*) and the timing of the tonic to burst transition. If *k* is larger, the tonic to burst transition occurs earlier (Figures 2G,H). I_D_ slowly inactivates in the model, over the course of seconds rather than milliseconds, to represent our hypothesis that I_D_ is non-inactivating in Purkinje neurons and that it reduces over time because of ion relaxation (*refer to Methods*). The burst to quiescent transition is produced by the hyperpolarising action of the Na^+^/K^+^ pump at the soma (*refer to Methods*). So, Na^+^/K^+^ pump activity sets the firing length (the length of the tonic & burst modes combined, labeled in Figure 2A).

**Figure 3:**
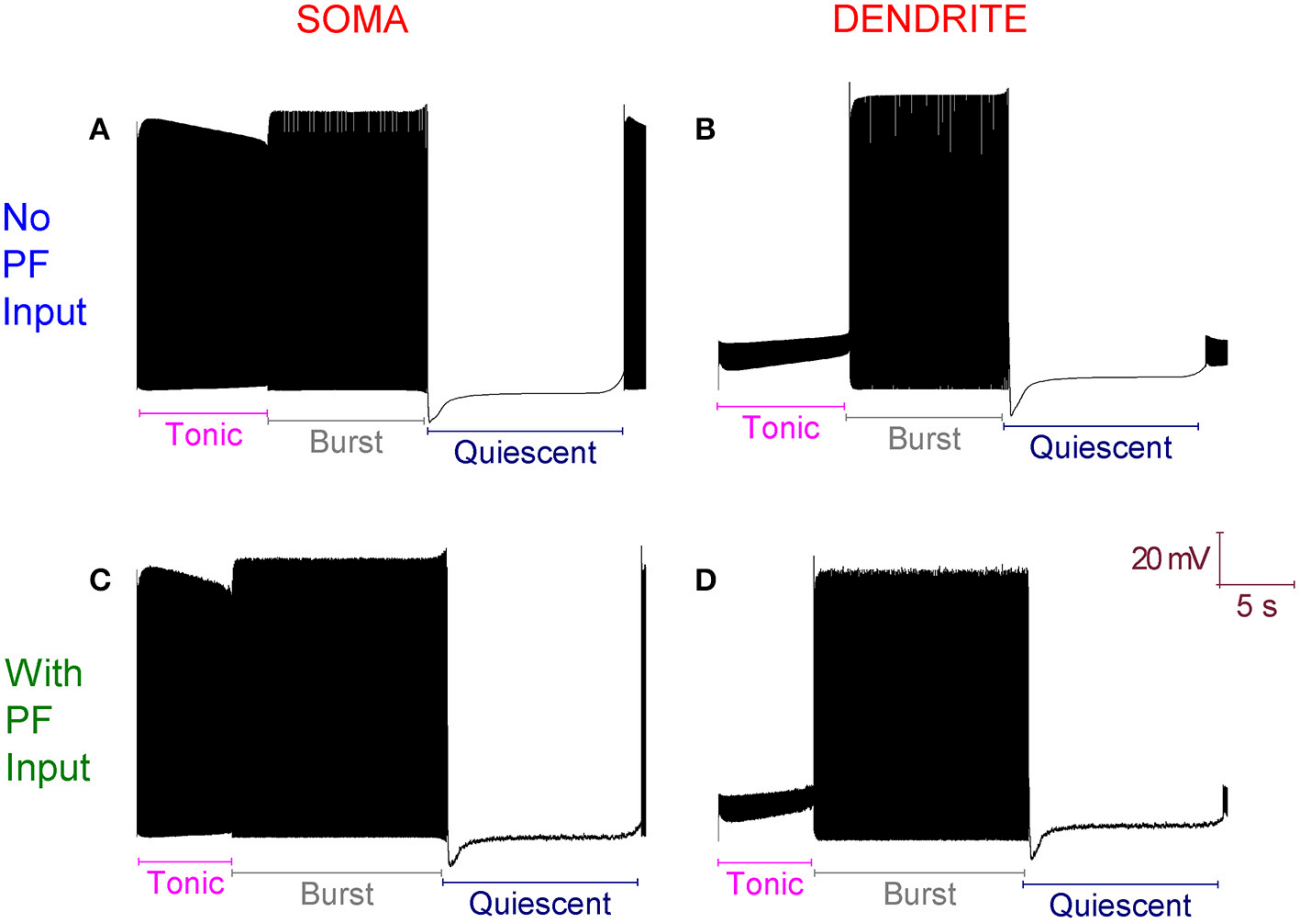
**With Parallel Fiber (PF) input, the Purkinje cell model still fires in the trimodal activity pattern. (A)** Without PF input: Single trimodal repeat, recorded at the soma, with the constituent tonic, burst and quiescent modes labeled. **(B)** The same trimodal repeat as **(A)** but recorded from a point within the dendritic tree. **(C)** With PF input: Single trimodal repeat, recorded at the soma, with the constituent tonic, burst and quiescent modes labeled. Comparing with **(A)**, the tonic mode is shorter when PF inputs are present. This is a function of a higher mean frequency of firing in the tonic mode, which produces an earlier tonic to burst mode transition. **(D)** The same trimodal repeat as **(C)** but recorded from a point within the dendritic tree. All panels are scaled by the same scale bar (20 mV, 5 s).

The introduction of excitatory PF inputs to the model dendrites does not change its activity motif. It still fires in the trimodal pattern (Figure 3). However, the mean frequency of firing in the tonic mode changes from 99 to 132 Hz. A 33% increase. This increased excitation causes the I_D_ excitability clamp to be overcome quicker and the tonic to burst transition to occur earlier.

### The purkinje cell model's intracellular Ca^2+^ dynamics permit it to replicate toggling

*In vitro* and *in vivo*, how does an identical, stereotypical CF input produce opposite transitions, toggling a Purkinje cell from [*down* → *up*] and [*up* → *down*] (Loewenstein et al., 2005; McKay et al., 2007)? This is an unresolved question.

**Figure 4:**
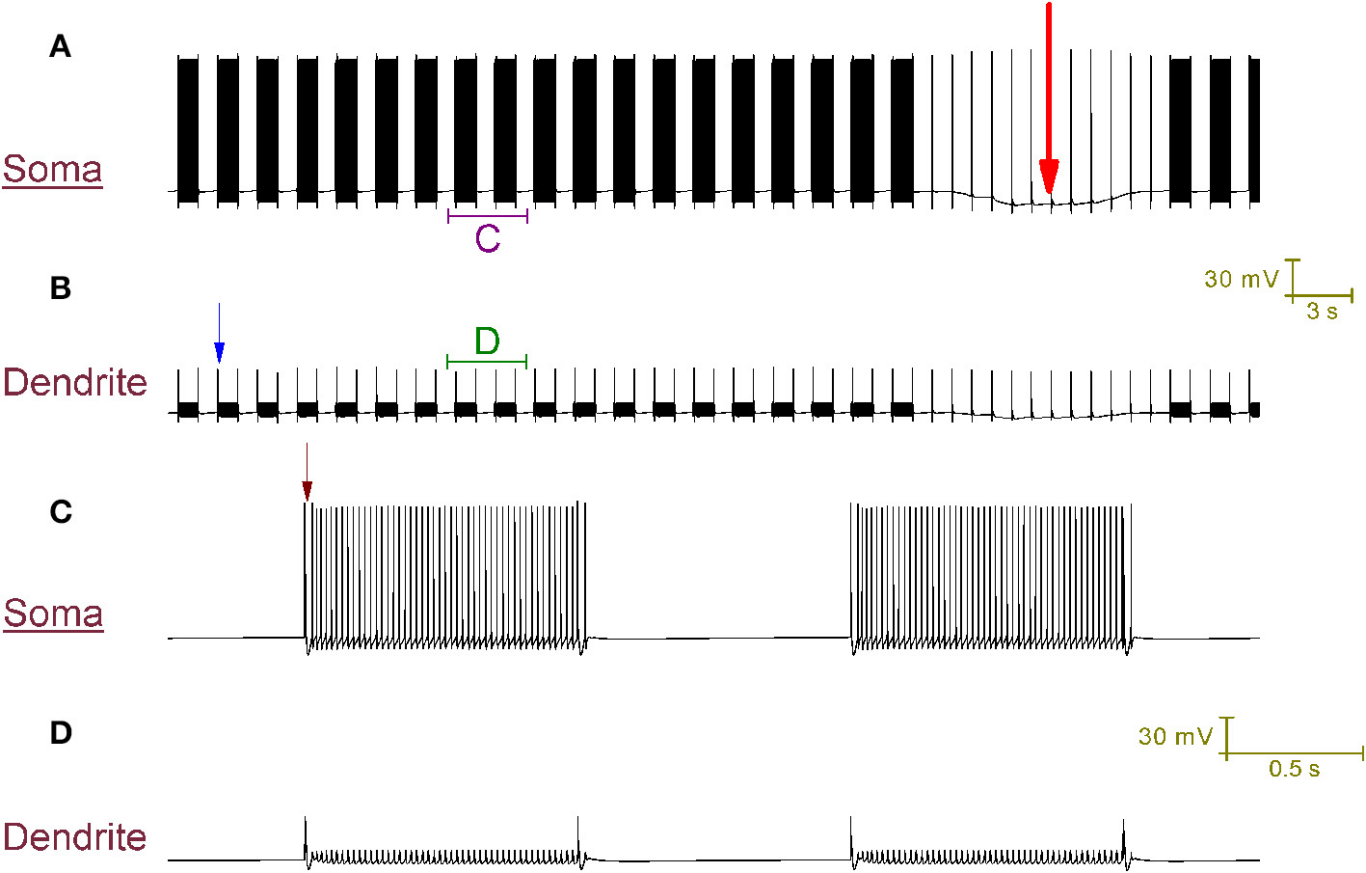
**CF input (1 Hz) can toggle the Purkinje cell model between a tonic firing (*up*) state and a quiescent (*down*) state. (A)** Somatic membrane potential (vs. Time). **(B)** Membrane potential at a dendritic location, over the same window of time as **(A)**. **(C)** Relates to the labeled part of **(A)**. **(D)** Relates to the labeled part of **(B)**. The scaling of **(A,B)** is encoded in the first scale bar (30 mV, 3 s). The scaling of **(C,D)** is encoded in the second scale bar (30 mV, 0.5 s). Every second, a CF input produces a large Ca^2+^ spike in the dendrites (one is labeled with a blue arrow in **B**) that generates a complex spike at the soma [distinguishable in the resolution of **(C)**; one is labeled with a brown arrow]. These CF input events toggle the cell between a tonic firing and a quiescent state at the frequency of CF input, 1 Hz **(A)**. When the cell is firing, a CF input toggles it to quiescence. When the cell is quiescent, a CF input toggles it to firing. A Na^+^/K^+^ pump generated silence can be observed in **(A)**, distinguishable from the other quiescent periods because it is much longer (~12 s as opposed to ~1 s) and is marked by a large red arrow. It is punctuated by CF driven spikes at the frequency of CF input (1 Hz); these CF inputs cannot evoke a state transition into the firing state over the course of this quiescence.

Our Purkinje cell model replicates toggling through mechanisms that we hypothesize to be responsible for toggling in real Purkinje cells (Figure 4). Every second a CF input to the model produces a large Ca^2+^ spike in the dendrites (one is labeled with a blue arrow in Figure 4B) that generates a complex spike at the soma (distinguishable in the resolution of Figure 4C; one is labeled with a brown arrow). These CF input events toggle the cell between a tonic firing and a quiescent state at the frequency of CF input, 1 Hz (Figure 4A). When the cell is firing, a CF input toggles it to quiescence. When the cell is quiescent, a CF input toggles it to firing. The significance of the longer quiescent period, labeled with the red arrow (Figure 4A), will be explained later on.

To our Purkinje cell model, CF input is both depolarising and hyperpolarising in parallel. It is depolarising because inward, depolarising cation flow through AMPA ionotropic receptors (AMPAR) opens voltage-gated Ca^2+^ channels that pass a depolarising Ca^2+^ influx into the dendrites. Yet, at the same time it is hyperpolarising because this Ca^2+^ activates hyperpolarising Ca^2+^-gated SK K^+^ channels in the dendrites. Which process is dominant dictates as to whether the CF input is *net* depolarising or hyperpolarising.

**Figure 5:**
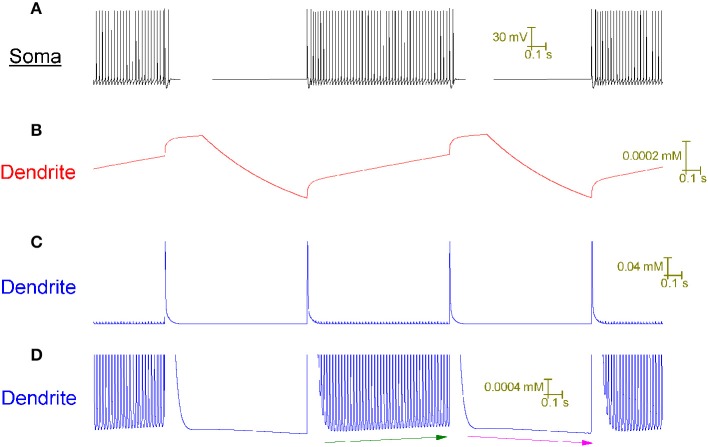
**The Purkinje cell model's capture of toggling behavior is made possible by its novel account of intracellular Ca^2+^ dynamics**. All panels relate to the same window of time. **(A)** Somatic membrane potential. **(B)** Model parameter *y* at a dendritic point. **(C)** Intracellular Ca^2+^ concentration, [Ca^2+^]_i_, at the same dendritic point as **(B)**. **(D)** Panel **(C)** at higher resolution. The model cell is toggled *up* (tonic firing) and *down* (quiescence) by CF input at a frequency of 1 Hz. Observe the cross-correlation between a Ca^2+^ spike event in the dendrites **(C)** and a toggle of state at the soma **(A)**. Model parameter *y* is the *set point* for the intracellular Ca^2+^ concentration ([Ca^2+^]_i_) i.e., the value that the system strives to return [Ca^2+^]_i_ to after a perturbation (*refer to Methods*). Parameter *y* rises during tonic firing; rises at a greater rate of change during a CF induced Ca^2+^ spike and decreases during quiescence **(B)**. These changes in *y* drive corresponding changes in [Ca^2+^]_i_—it rises during tonic firing, rises at a greater rate of change during a CF induced Ca^2+^ spike and decreases during quiescence **(C,D)**. The [Ca^2+^]_i_ increase during tonic firing, and decrease during quiescence, is clear in the high resolution of **(D)** where it is highlighted by green and purple arrows respectively. During tonic firing, one can see that the rise in [Ca^2+^]_i_ is due to a rising set point value (*y*) because the [Ca^2+^]_i_ value that the system is returned to after a calcium spike event increases over time (refer green line).

In the model, the tonic firing (*up*) state responds differently to CF input than the quiescent (*down*) state because it has a different intracellular Ca^2+^ concentration in its dendrites (Figure 5). During tonic firing, intracellular Ca^2+^ accumulates as a function of voltage-gated Ca^2+^ entry. During quiescence it recedes as Ca^2+^ extrusion exceeds any remaining Ca^2+^ entry. The firing state has a higher [Ca^2+^]_i_ level in its dendrites than the quiescent state.

During the firing state, the CF conferred rise in [Ca^2+^]_i_—added to the firing state's higher basal [Ca^2+^]_i_ level—stimulates sufficient hyperpolarising current flow (through Ca^2+^-gated SK K^+^ channels) to produce a *net* hyperpolarisation and the cell is toggled to quiescence. During the quiescent state, the CF conferred rise in [Ca^2+^]_i_—added to the quiescent state's lower basal [Ca^2+^]_i_ level—cannot confer sufficient hyperpolarising current flow (through Ca^2+^-gated SK K^+^ channels) to outweigh the depolarising effect of the Ca^2+^ entry; there is a *net* depolarisation and the cell is toggled to firing.

In this way, the same stereotypical CF input produces different outcomes, depending on the prior state of the model cell i.e., the CF input acts as a *toggle* switch, toggling the model cell from whichever of the two states it is in to the alternate state.

Note that with a CF conferred *down* to *up* transition, after the CF conferred complex spike event and before the onset of tonic firing, there is a short pause (Figure 4) which is a detail observed in experimental data (McKay et al., 2007). The capture of this pause is a function of, and provides some validation to, the model's intracellular Ca^2+^ dynamics.

### CF input can produce a tonic firing pattern punctuated by complex spikes and short pauses in the purkinje cell model

**Figure 6:**
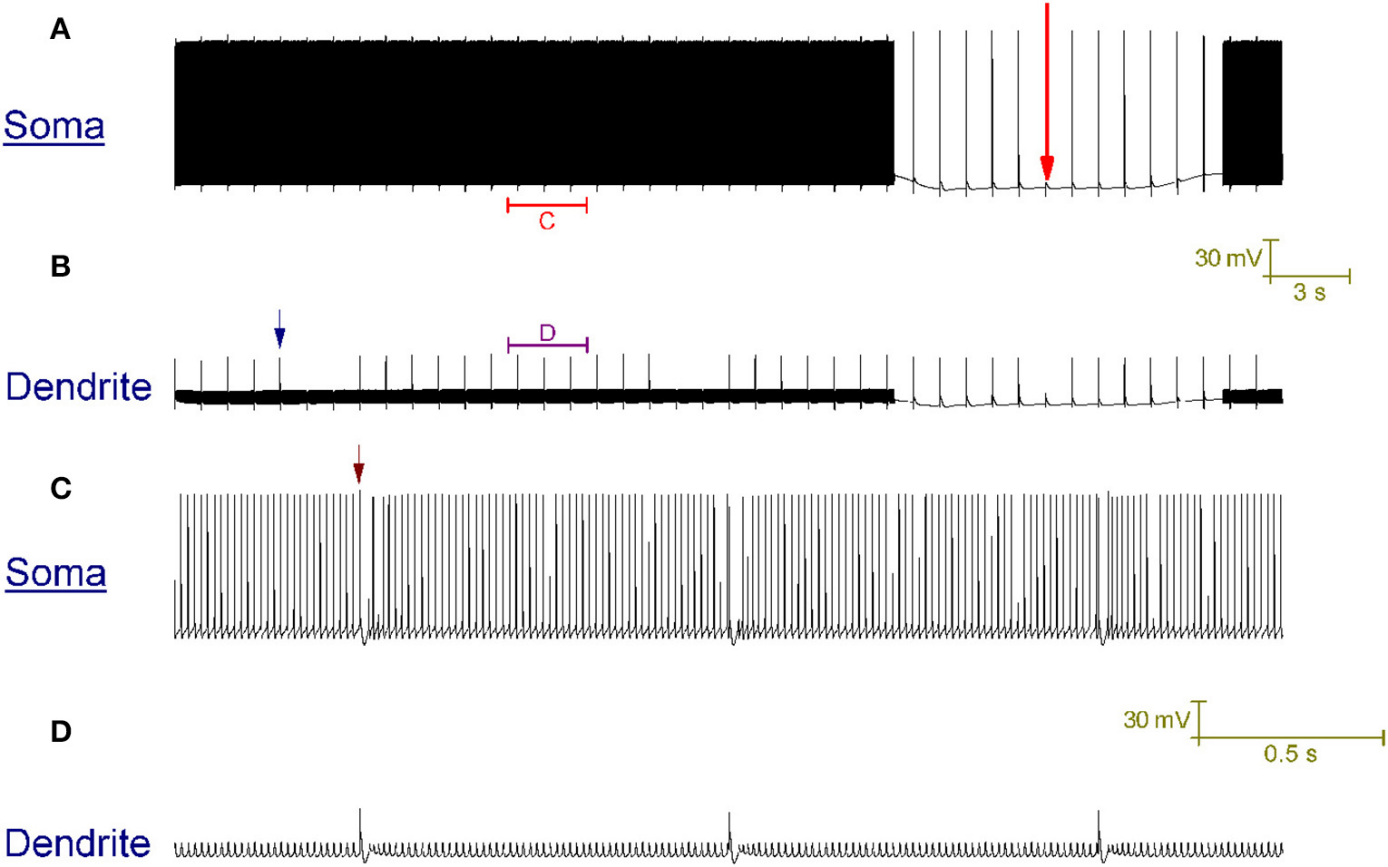
**CF input (1 Hz) can produce a tonic firing pattern punctuated by complex spikes and short pauses. (A)** Somatic membrane potential (vs. Time). **(B)** Membrane potential at a dendritic location; for the same window of time as **(A)**. **(C)** Relates to the labeled part of **(A)**. **(D)** Relates to the labeled part of **(B)**. The scaling of **(A,B)** is encoded in the first scale bar (30 mV, 3 s). The scaling of **(C,D)** is encoded in the second scale bar (30 mV, 0.5 s). Every second a CF input produces a large Ca^2+^ spike in the dendrites [one is labeled with a blue arrow in **(B)**] that generates a complex spike at the soma, followed by a short evoked after-pause [distinguishable in the resolution of **(C)**; one is labeled with a brown arrow]. The model generates this pattern in response to CF input, as opposed to the toggled pattern of Figure 4, when the model parameter g_sk_ (*refer to Methods*) is 0.62 S/cm^2^ as opposed to 0.72 S/cm^2^. A Na^+^/K^+^ pump generated silence can be observed in **(A)**, marked by a large red arrow. It is punctuated by CF driven spikes at the frequency of CF input (1 Hz).

*In vitro*, repetitive CF input (~1 Hz) doesn't always switch a trimodal Purkinje cell to bimodal patterning. In some cases, it switches the cell to a tonic firing pattern that is punctuated, at the frequency of CF input, by a complex spike and its short evoked after-pause (~20 ms long) (McKay et al., 2007). In our model, CF input generates this pattern (Figure 6), as opposed to the CF driven bimodal patterning, when the SK maximal conductance (*g*_sk_) is 0.62 S/cm^2^ as opposed to 0.72 S/cm^2^. The after-pause is a function of the model's intracellular Ca^2+^ dynamics.

### CF input blocks the bursting mode, but not the quiescent mode, of the trimodal firing pattern

*In vitro*, repetitive CF input (1 Hz) blocks the trimodal firing pattern and replaces it with a tonic firing pattern, interrupted either by quiescent periods (~1 s long) or short pauses (~20 ms long) (McKay et al., 2007). No bursting mode is observed. However, in both these firing patterns there are very long quiescent periods (>>1 s), where the only deflections in somatic membrane potential are attributable to CF driven spikes, at the frequency of CF input, and in which CF input cannot evoke a state transition into the firing state (Figure 1D of McKay et al., 2007). The cause of these long quiescent periods is not known.

We hypothesize that they are the enduring quiescent mode of the blocked trimodal firing pattern, which we propose is generated by electrogenic Na^+^/K^+^ pumping. So, we suggest that although CF input blocks the trimodal pattern's bursting mode, it does not block its quiescent mode. Indeed, the incorporated Na^+^/K^+^ pump system enables the model to replicate these long quiescent periods in the CF produced tonic firing patterns (Figures 4, 6, labeled with a red arrow).

So, we tentatively suggest that there are 2 different classes of *long* quiescent period in Purkinje cell activity: (1) CF conferred [~1 s long]; (2) Na^+^/K^+^ pump conferred [>>1 s long]. The former is regulated by intracellular Ca^2+^ dynamics, the latter by intracellular Na^+^ dynamics. In addition, there can be *short* quiescent periods: short pauses after complex spikes (~20 ms long), which are a function of intracellular Ca^2+^ dynamics.

### In the purkinje cell model, CF input decreases the mean frequency of firing to a range where PF input can greatly increase it, setting the gain of the PF response

*In vitro*, PF input increases tonic firing frequency. CF input decreases it. In fact, CF input decreases the mean frequency of firing to a range where PF input can greatly increase it, setting the *gain* of the PF response (McKay et al., 2007). Our model replicates this *gain* computation. In the model, with the introduction of just PF inputs, the mean frequency of tonic firing shifts from 99 to 132 Hz—a 33% increase. With the introduction of just CF input (*g*_sk_ = 0.62 S/cm^2^), the mean frequency of tonic firing shifts from 99 to 54 Hz—a 55% decrease. With CF input already introduced and then with the subsequent introduction of PF inputs, the mean frequency of tonic firing shifts from 54 to 80 Hz—a 68% increase. So, CF input confers a *gain* in the PF induced frequency change.

### I_h_ activity controls purkinje cell, and purkinje model, activity

**Figure 7:**
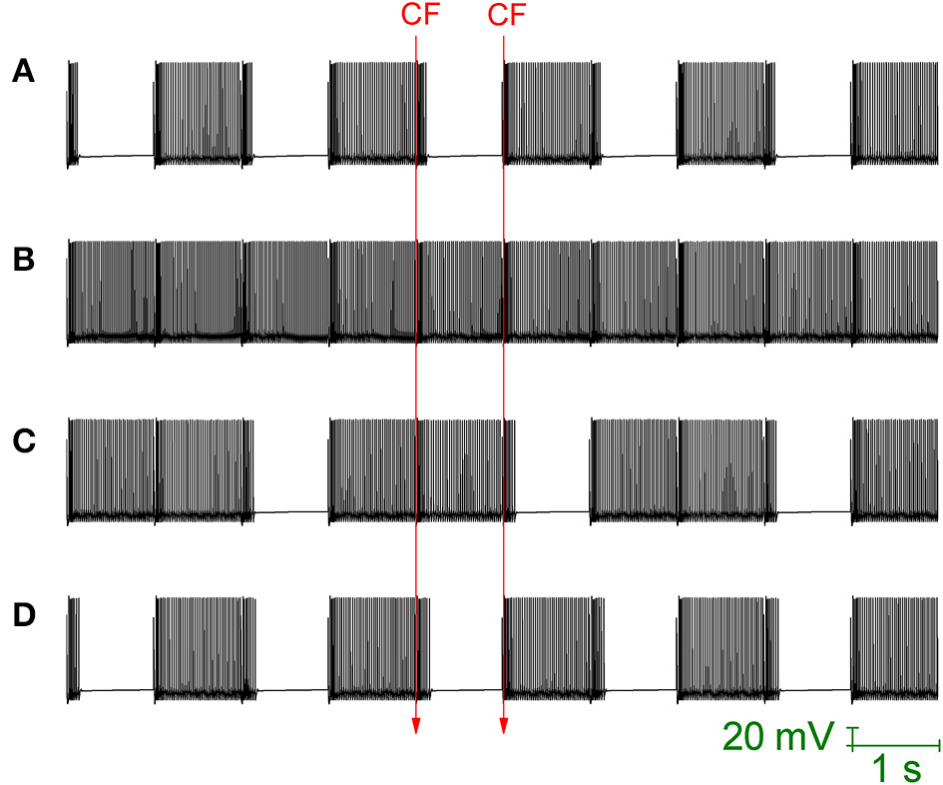
**I_h_ activity can mask Purkinje cell bistability**. All panels relate to the Purkinje cell model's somatic membrane potential (vs. Time). The model is innervated with CF input at a frequency of 1 Hz (i.e., at 1 second intervals). The timing of two representative CF input events is shown across the panels which are all scaled by the same scale bar (20 mV, 1 s). **(A)** With I_h_ conductance density set to 0 (to replicate a pharmacological block of I_h_), CF input (1 Hz) can still *toggle* the Purkinje cell model between a tonic firing (*up*) state and a quiescent (*down*) state i.e., the model's toggled, bistable behavior is not conditional upon I_h_ activity. **(B)** Raising the model's I_h_ conductance density at the soma, from its default value of 1.04–5 mS/cm^2^, results in CF input being unable to *toggle* the Purkinje cell state. Instead, CF input produces a continuous tonic firing pattern, interrupted at the frequency of CF input (1 Hz) by complex spikes and their short evoked after pauses (i.e., the activity pattern of Figure 6) i.e., increased I_h_ activity can block/mask Purkinje cell bistabilty. **(C)** With a raised I_h_ conductance density blocking the bistable pattern, a compensatory raise in the g_sk_ model parameter (SK conductance density) can rescue and reinstate the bistable pattern. In this panel, g_sk_ is raised from 0.72–0.85 S/cm^2^ which is not quite enough and a mixed pattern of continuous/bistable is expressed. **(D)** In this panel, g_sk_ is raised higher (0.88 S/cm^2^) and the bistable pattern is reinstated fully.

Loewenstein et al. (2005) produced a Purkinje cell model that places the hyperpolarisation activated cation current (I_h_) as the critical conductance to Purkinje cell bistability. This model is heavily reduced, with no spiking dynamics and the bistability is between two different rest states (hyperpolarized and depolarized). The model cannot account for the fact that I_h_ block by ZD 7288, or I_h_ downregulation by serotonin, does not abolish bistability (Williams et al., 2002; Fernandez et al., 2007). Indeed, I_h_ block/downregulation has been observed to increase the observation of bistability (to “unmask” it; Williams et al., 2002). The bistability of our Purkinje cell model is not I_h_ dependent and is maintained when this conductance is removed, to simulate the application of an I_h_ blocking drug (Figure 7A). So, the model does not simply replicate the CF toggled bistable pattern, but can replicate a pharmacological challenge to it.

When the maximal conductance of the I_h_ current, g_ih_, is increased in the model's soma (from it's default of 1.04 mS/cm^2^, a value inherited from the model's origin in Forrest et al., 2012, to 5 mS/cm^2^) then the CF toggled bistable pattern is “masked” by a pattern of continuous tonic firing, punctuated by complex spike events and their short after-pauses at the frequency of CF input (Figure 7B). However, although CF toggled quiescent periods are eradicated, Na^+^/K^+^ pump generated silences still occur. This is the same activity pattern as reported in Figure 6. So, increased I_h_ activity can block/mask Purkinje cell bistabilty. With a raised g_ih_ value blocking the bistable pattern, a compensatory raise in the g_sk_ model parameter (SK maximal conductance) can rescue and reinstate the bistable pattern (Figures 7C,D).

So, *in vitro*, CF input can switch an intrinsically trimodal Purkinje cell into two different, alternative activity patterns: [A] toggled bistability, or [B] continuous tonic firing with punctuations of complex spikes and after pauses (McKay et al., 2007). In our model, which one is expressed can be set by the ratio of somatic I_h_ activity to dendritic SK activity. I_h_ activity has been shown to be regulated by serotonin in Purkinje cells and it may be a molecular switch between these patterns (Williams et al., 2002). In addition, SK has been shown to be under transmitter control in many types of neurons (Nicoll, 1988). Interestingly, *in vivo*, electrical stimulation of the Raphe complex releases serotonin and induces long pauses in Purkinje cell output (Strahlendorf et al., 1979; Weiss and Pellet, 1982), consistent with the ability for serotonin to increase toggling behavior and affect locomotor activity (Mendlin et al., 1996).

## Discussion

### The purkinje cell model reconciles *in vitro* and *in vivo* behavior

The Purkinje cell's trimodal firing pattern is observed in the *in vitro* slice preparation when its synaptic inputs are compromised (by the slicing plane or pharmacological block) (Womack and Khodakhah, 2002, 2003, 2004; Womack et al., 2004; McKay and Turner, 2005; McKay et al., 2007; Forrest et al., 2012). In our modeling, we show a possible mechanistic relation between the trimodal firing pattern and the CF toggled bimodal pattern. We give an account of how, without synaptic input, Purkinje cells can spontaneously fire in a trimodal pattern and how CF input, at a physiological frequency, can switch them to a CF toggled bimodal pattern. In this way we are reconciling how the trimodal pattern might correspond to more physiological patterns of activity. This is useful because a lot of research is, and has been, conducted on the trimodal firing pattern (Womack and Khodakhah, 2002, 2003, 2004; Womack et al., 2004; McKay and Turner, 2005; McKay et al., 2007; Forrest et al., 2012). This work can be served well by showing how it relates to patterns that have been observed *in vivo*. Whilst it is true that a trimodal-like oscillatory pattern can emerge *in vivo*, this has only been reported when CF input is silenced by a lesion of, or injection of lignocaine into, the inferior olive (Cerminara and Rawson, 2004). So, the direct physiological relevance of it is up for debate. However, its study can still add insight so long as we understand how its activity relates to activity patterns in the behaving animal.

### The purkinje cell model can perform *toggle* and gain computations upon its inputs

In the Purkinje cell model, *toggle* and *gain* computations hinge on underlying Ca^2+^ dynamics. We hypothesize that this is the case for real Purkinje neurons.

The model's intracellular Ca^2+^ concentration—[Ca^2+^]_i_—provides a short-term memory store. It records a history of firing and inputs, to dictate how the model cell responds to future inputs. For example, [Ca^2+^]_i_ memorizes a CF input, which causes the Purkinje cell model to then respond differently to a PF input than it would otherwise (CF input increases the *gain* of the response to PF input). This Ca^2+^ memory has a lifespan and once it has expired the model cell responds by default. Unless there is another CF input to renew and perpetuate the memory setting.

So, we hypothesize that the membrane potential (*V)* is not the Purkinje cell's only coding variable. We hypothesize that [Ca^2+^]_i_ is a coding variable as well. These two interact, with the Ca^2+^ memory being encoded and decoded by the membrane potential. Encoding is by way of CF input causing membrane depolarisation, which then opens voltage-gated Ca^2+^ channels to raise [Ca^2+^]_i_. Decoding is by way of [Ca^2+^]_i_ modulating the membrane potential via Ca^2+^-activated K^+^ channels.

CF input, in interaction with the [Ca^2+^]_i_ memory store, can dictate the timing and duration of quiescent periods (*toggling*) in the Purkinje cell model. This system might be modulated in the physiological setting by signaling cascades, which have been shown to regulate intracellular Ca^2+^ dynamics (Falcke and Malchow, 2003).

### Neural coding

Spikes are frequently taken as the basic unit of neural coding. However, silences in spiking may be just as meaningful for the Purkinje cell as its firing output is inhibitory to the downstream deep cerebellar nuclei (DCN). So a pause in its spiking would convey disinhibition (Jaeger, 2007; Steuber et al., 2007). We speculate that the timing and duration of quiescent periods might be salient to how the Purkinje cell encodes information.

### CF input blocks the bursting mode, but not the quiescent mode, of the trimodal firing pattern

*In vitro*, a physiological frequency of CF input (~1 Hz) switches Purkinje cells out of the trimodal firing pattern and into a nonbursting pattern of activity. On this basis it is probable that the trimodal firing pattern is not applicable *in vivo*. However, in these experiments, although CF input blocks the trimodal pattern's bursting mode, long (>>1 s) quiescent periods can still be observed. We hypothesize that these are the enduring quiescent mode of the trimodal pattern, generated by electrogenic Na^+^/K^+^ pumping. On this basis, we suggest that Na^+^/K^+^ pump generated silences occur physiologically. Furthermore, we propose that the intracellular Na^+^ concentration ([Na^+^]_i_) acts as a memory element in the Purkinje cell. It memorizes firing history and sets Na^+^/K^+^ pump activity to dictate the timing and duration of quiescent periods. This action could be externally and internally regulated: the Na^+^/K^+^ pump is a receptor for the endo-ouabain signaling molecule (Xie and Cai, 2003) and Na^+^/K^+^ pump activity might be modulated by intracellular signaling cascades (Therien and Blostein, 2000; Bagrov and Shapiro, 2008). Relevantly, a mutation in the Na^+^/K^+^ pump causes rapid-onset dystonia-parkinsonism (RDP), which has symptoms to indicate that it is a pathology of cerebellar computation (Cannon, 2004; de Carvalho et al., 2004). Indeed, there is a growing body of work showing that Na^+^/K^+^ pumps might subserve information processing roles in neurons (Arganda et al., 2007; Scuri et al., 2007; Forrest, 2008; Forrest et al., 2009, 2012; Pulver and Griffith, 2010; Zhang and Sillar, 2012). For cerebellar Purkinje cells, we speculate that the Na^+^/K^+^ pump is not simply a homeostatic mechanism to ionic gradients; we venture that it is a computational element. In experimental support, an ouabain block of Na^+^/K^+^ pumps in the cerebellum of a live mouse results in it displaying ataxia and dystonic like postures (Calderon et al., 2011). If Na^+^/K^+^ pumps are computational entities in the cerebellum, it will change present estimates of the metabolic cost to neural information (where ionic pumping is considered as purely cost) (Laughlin et al., 1998).

Alcohol consumption corrupts motor function and this is a factor in a large number of accidental injuries and deaths every year. Alcohol has been shown to modulate Na^+^/K^+^ pumping in preparations from rodent brains (Foley and Rhoads, 1994; Ledig et al., 1985; Syapin et al., 1985). It would be interesting to investigate if alcohol inhibits Na^+^/K^+^ pumping in cerebellar Purkinje neurons, and whether this is a factor in the motor dysfunction concordant with inebriation.

### Ion to network computation

We propose that the cerebellar Purkinje cell has two memory stores in parallel: [Ca^2+^]_i_ and [Na^+^]_i_. They both can regulate the timing and duration of quiescent periods, via different mechanisms, and we speculate that these silences have a function. These two memory stores may cross-talk through the action of the Na^+^/Ca^2+^ exchanger. Looking at the frequency of CF prompted silences, via Ca^2+^ computation, and the lesser frequency (but longer length) of Na^+^/K^+^ conferred silences, via Na^+^ computation, we could propose that these two systems are specialized for, and operating on, different time scales.

Our view is that these slow processes (on the scale of seconds and minutes) are relevant and computationally advantageous because, by conferring an access to longer time scales, they permit storage and short-term processing of sensory information in the cerebellar cortex. To elaborate, they permit different dynamical states to be sustained in the cerebellar cortex for extended periods. Each of these states is associated with a specific configuration of *up* and *down* states in different Purkinje cells. These network states could store information and perform computations. So, these network computations sit upon the proposed intracellular ion computations (Na^+^, Ca^2+^) that dictate the activity state of individual Purkinje neurons.

### Purkinje cell inputs converge upon the deep cerebellar nuclei (DCN)

Purkinje cells provide inhibitory input to the Deep Cerebellar Nuclei (DCN). Long quiescent periods may be important in cerebellar functioning because numerous Purkinje neurons converge upon—and inhibit—a single DCN neuron (~40:1). If all these Purkinje neurons are continually, simultaneously active then it might be that the DCN neuron is unable to fire in any meaningful way.

With a proportion of the Purkinje cells quiescent, only a fraction of the population is active and relevant. The members making up this relevant sub-set can be switched and changed in a controlled fashion, which can be utilized as a computational feature.

### Confusion in the literature

The ability to detect Purkinje cell toggling, by CF input, has been examined *in vivo* with anesthetized and awake animals. Toggling is observable in anesthetized animals (Loewenstein et al., 2005; Schonewille et al., 2006; Kitamura and Hausser, 2011). But some have concluded that this toggling is an experimental artifact, produced by the use of anesthetic, and that toggling cannot be observed in awake animals (Schonewille et al., 2006). However, the validity of this latter study has been challenged (Loewenstein et al., 2006) and Purkinje cell toggling has since been reported in awake cats (Yartsev et al., 2009). For a review of this issue refer to Engbers et al. (2013). In our interpretation of the contemporary literature, we think that Purkinje cell toggling is likely to be a physiological phenomenon.

### An alternative model of purkinje cell toggling

Fernandez et al. have published a 5 equation model of Purkinje cell toggling by CF input (Fernandez et al., 2007). This model is heavily reduced as compared to the model of this study. It only has four voltage-gated conductances, with a limited parameterisation to experimental data, and no synapses, pumps or exchangers. It cannot fire in the trimodal pattern with its repeating motif of tonic, burst and quiescent phases. Indeed, it can only fire upon an external input and does not capture the spontaneous foundation to Purkinje cell electrical activity. It does not include the resurgent Na^+^ current, which may be an important factor in the *down* to *up* transition (Fernandez et al., 2007). Their model's *up* to *down* transition is dependent upon a *slow* K^+^ current in the dendrite, which Fernandez et al. suggest could arise from an interaction between internal Ca^2+^ and Ca^2+^-activated K^+^ channels. Our model represents this latter possibility much more explicitly. Both models have an I_h_ current that acts against the observation of bistability. This in contrast to the model of Loewenstein et al. (2005), where I_h_ is *the* critical conductance to Purkinje cell bistability. However, as discussed in our Results, this model cannot reconcile with experiments where I_*h*_ block actually increases the observation of bistability. Our model and that of Fernandez et al. complement one another. We capture more of the physiology. But their model, being simpler and less computationally intensive, is much more tractable to analysis. In fact, in their paper, they go on to report a 2 equation model of the Purkinje cell which is even more tractable. But this, of course, is at a further cost to biological fidelity.

### The future

The model should be modified to capture a wider range of Purkinje cell activity, whilst ensuring that it retains the behaviors detailed here. For example, our model can fire Ca^2+^ spikes in the dendrites, which travel to the soma and drive a somatic bursting pattern. Experimental (Swensen and Bean, 2003) and modeling (Forrest, 2013) research suggests that, in addition to this dendritic mechanism to somatic bursting, the soma can intrinsically burst of its own accord. At present our model cannot capture this behavior. If it can be modified to do so, without any loss of present functionality, this will be an improved model version. In this way, we hope that the model will proceed through a series of iterations each better than the last.

### Summary

In our model, we have identified different classes of Purkinje cell quiescence. Firstly, short pauses after complex spikes (~20 ms long) and, secondly, CF toggled quiescent periods (~1 s long); both a function of intracellular Ca^2+^ dynamics. I_h_ activity (shown to be regulated by serotonin; Williams et al., 2002) can dictate which of these is expressed. In addition, there are more infrequent, longer quiescent periods (>>1 s) produced by the electrogenic action of the Na^+^/K^+^ pump, as a function of of intracellular Na^+^ dynamics. We hypothesize that these quiescent forms correspond to behavior in real Purkinje cells, and that they have a coding role in the Purkinje cell relay to the DCN. We suggest that the Na^+^/K^+^ pump is directly involved in the information processing performed by the cerebellar Purkinje cell.

## Funding

The author conducted this work in the period 2006–2007, whilst doing his Ph.D. degree at the University of Warwick. He was funded at this time by the Medical Research Council (MRC) of the UK, http://www.mrc.ac.uk/index.htm. The funders had no role in study design, data collection and analysis, decision to publish, or preparation of the manuscript. The author declares that no competing interests exist. This work was reviewed by the author, and written up for journal publication, in 2012 (with no funding appropriated for this task). The author had previously presented his findings as a component of a poster presentation in 2009: Forrest MD, Wall MJ, Press DA. The Sodium-Potassium Pump Controls the Intrinsic Firing of the Cerebellar Purkinje Neuron. Poster session presented at: 16th International Conference on Neural Information Processing; 2009 Dec 1–5; Bangkok, Thailand.

### Conflict of interest statement

The author declares that the research was conducted in the absence of any commercial or financial relationships that could be construed as a potential conflict of interest.
